# Nitric Oxide Functions as a Downstream Signal for Melatonin-Induced Cold Tolerance in Cucumber Seedlings

**DOI:** 10.3389/fpls.2021.686545

**Published:** 2021-07-23

**Authors:** Yiqing Feng, Xin Fu, Lujie Han, Chenxiao Xu, Chaoyue Liu, Huangai Bi, Xizhen Ai

**Affiliations:** State Key Laboratory of Crop Biology, Key Laboratory of Crop Biology and Genetic Improvement of Horticultural Crops in Huanghuai Region, College of Horticulture Science and Engineering, Shandong Agricultural University, Tai'an, China

**Keywords:** melatonin, nitric oxide, antioxidant system, CO_2_ assimilation, photoprotection, cold stress, signal pathway

## Abstract

Melatonin (MT) and nitric oxide (NO) are two multifunctional signaling molecules that are involved in the response of plants to abiotic stresses. However, how MT and NO synergize in response to cold stress affecting plants is still not clear. In this study, we found that endogenous MT accumulation under cold stress was positively correlated with cold tolerance in different varieties of cucumber seedlings. The data presented here also provide evidence that endogenous NO is involved in the response to cold stress. About 100 μM MT significantly increased the nitrate reductase (NR) activity, *NR*-relative messenger RNA (mRNA) expression, and endogenous NO accumulation in cucumber seedlings. However, 75 μM sodium nitroprusside (SNP, a NO donor) showed no significant effect on the relative mRNA expression of tryptophan decarboxylase (*TDC*), tryptamine-5-hydroxylase (*T5H*), serotonin-N-acetyltransferase (*SNAT*), or acetylserotonin O-methyltransferase (*ASMT*), the key genes for MT synthesis and endogenous MT levels. Compared with H_2_O treatment, both MT and SNP decreased electrolyte leakage (EL), malondialdehyde (MDA), and reactive oxygen species (ROS) accumulation by activating the antioxidant system and consequently mitigated cold damage in cucumber seedlings. MT and SNP also enhanced photosynthetic carbon assimilation, which was mainly attributed to an increase in the activity and mRNA expression of the key enzymes in the Calvin–Benson cycle. Simultaneously, MT- and SNP-induced photoprotection for both photosystem II (PSII) and photosystem I (PSI) in cucumber seedlings, by stimulating the PsbA (D1) protein repair pathway and ferredoxin-mediated NADP^+^ photoreduction, respectively. Moreover, exogenous MT and SNP markedly upregulated the expression of chilling response genes, such as inducer of *CBF* expression (*ICE1*), C-repeat-binding factor (*CBF1*), and cold-responsive (*COR47*). MT-induced cold tolerance was suppressed by 2-(4-carboxyphenyl)-4,4,5,5-tetramethylimidazoline-1-oxyl-3-oxide (cPTIO, a specific scavenger of NO). However, p-chlorophenylalanine (p-CPA, a MT synthesis inhibitor) did not affect NO-induced cold tolerance. Thus, novel results suggest that NO acts as a downstream signal in the MT-induced plant tolerance to cold stress.

## Introduction

Cucumbers (*Cucumis sativus* L.) are sensitive to cold stress and are mainly cultivated through the winter in solar greenhouses in northern China; therefore, they often encounter cold stress. Cold stress adversely affects photosynthesis by damaging the electron transfer chain in chloroplasts and mitochondria, which naturally results in excessive reactive oxygen species (ROS) (Fan et al., 2015), and has become a major limitation to crop productivity and quality. It has been reported that plants can improve their cold tolerance to ensure growth and development mainly through stimulating antioxidant system activity and osmotic adjusting ability (Pan et al., 2020). Recently, a growing number of evidence suggest that various plant hormones, such as jasmonic acid (JA), brassinosteroids (BRs), abscisic acid (ABA), and salicylic acid (SA) (Kagale et al., 2007; Hu et al., 2013; Zhang et al., 2014; Ding et al., 2015), are also involved in responses to cold stress and regulating plant cold tolerance.

Melatonin (MT) is a small indoleamine molecule that plays a prominent role in regulating various physiological processes, including seed germination, plant growth, flower development, and root system structure (Murch and Saxena, 2002; Hernández-ruiz et al., 2005; Wang et al., 2012; Arnao, 2014). In recent years, many reports have emphasized the importance of MT in response to abiotic stresses, such as extreme temperature, drought, osmotic stress, salinityalkalinity, heavy metals (Cui et al., 2017, 2018; Ahammed et al., 2018; Fan et al., 2018; Nawaz et al., 2018; Qi et al., 2018; Yan et al., 2019; Kong et al., 2020; Siddiqui et al., 2020). To date, almost all studies consider that MT plays a critical role in ROS scavenging, either as a stress-induced agent or a protective molecule (Arnao and Hernandez-Ruiz, 2015; Erland et al., 2018; Martinez et al., 2018; Li et al., 2021). Additionally, reliable evidence suggests that MT achieves its antioxidant capacity through direct detoxification of ROS and reactive nitrogen species and indirect stimulation of antioxidant enzymes (Wang et al., 2017). Ahammed et al. (2018) found that MT-deficient caffeic acid O-methyltransferase1 (*COMT1*)-silenced tomato plants were more sensitive to heat-induced oxidative stress, and exogenous MT-pretreated plants showed an increase in the activity and messenger RNA (mRNA) expression of antioxidant enzymes in *COMT1*-silenced plants. Al-Huqail et al. (2020) suggested that MT induced plant defense mechanisms by enhancing Pro, TSCs, TPC, nutrient (N and P) uptake, and enzymatic and non-enzymatic antioxidants. Li et al. (2019) demonstrated that MT alleviates abiotic stress-induced damage in tea plants by scavenging ROS and regulating antioxidant systems. Recently, some evidence has indicated that MT, acting as a phytohormone-like molecule or secondary messenger, participates in many signaling events, including the response to abiotic stress in plants (Yan et al., 2019). MT appears to act as a key molecule in the plant immune response, together with other well-known molecules, such as nitric oxide (NO), and hormones, such as JA and SA (Arnao and Hernandez-Ruiz, 2018).

Previous studies have shown that NO, an important endogenous gas signaling molecule, is involved in almost all biological processes in plants, including seed germination (Wimalasekera et al., 2011), plant maturation, and senescence (Guo and Crawford, 2005; Mishina et al., 2007). Additionally, NO has been shown to regulate numerous plant responses to a variety of biotic and abiotic stresses and to relieve the damage caused by oxidative stress (Siddiqui et al., 2011; Ahanger et al., 2020; Kaya et al., 2020b,c; Singh et al., 2020). According to previous studies, some shared physiological functions of MT and NO have been verified in their regulation of plant tolerance to stress conditions; in particular, both are involved in ROS signaling pathways in plants (Park et al., 2013; Scheler et al., 2013; Kaya et al., 2019, 2020a).

Recently, it was reported that MT can increase the resistance of plants to biotic stress *via* the NO-mediated signaling pathway (Shi H. T. et al., 2015). Exogenous MT increased NO accumulation in alkaline-stressed tomato roots, but exogenous NO showed little effect on MT content, suggesting that NO, acting as a downstream signal, is involved in MT-induced alkaline tolerance in tomatoes (Liu et al., 2015). However, limited information has been provided about the relationship between MT and NO in plants under cold stress, and the physiological and molecular mechanisms of interaction between MT and NO during detoxifying cold stress are still unclear. Therefore, in this study, we investigated the interrelationship between MT and NO as well as their role in ROS scavenging, CO_2_ assimilation, and photoprotection under cold stress. Our research aimed to elucidate the mechanism of the effects of MT and its signaling pathways in the cold stress response of cucumbers.

## Materials and Methods

### Plant Materials and Treatments

Cucumber [*C. sativus* L. “Jinyou 35” (JY35) and “Zhongnong 6” (ZN6)] seeds were soaked in water for 6 h, followed by incubation on moistened filter papers in the dark for 24 h at 28°C for germination. The germinated seeds were sown in a growth medium, which consisted of peat, vermiculite, and perlite (5:3:1, v/v) in a climate chamber. The growth environment conditions were maintained as follows: 25/18°C day/night temperature, 80% relative humidity, and 11 h photoperiod with photon flux density (PFD) of 600 μmol m^−2^·s^−1^.

### Chilling and MT or NO Treatment

To examine the effect of MT and sodium nitroprusside (SNP, a NO donor) on cold tolerance of cucumber, seedlings with three leaves were foliar sprayed with 0 (control), 25, 50, 75, 100, or 125 μM MT or pretreated with 0 (control), 25, 50, 75, 100, or 125 μM SNP (10 ml per plant). At 24 h after pretreatment with MT or SNP, the seedlings were exposed to 5°C for 72 h for the analysis of malondialdehyde (MDA) content and electrolyte leakage (EL). The seedlings were pretreated with 100 μM MT, 75 μM SNP, or distilled water (control) for examining the effect of MT on endogenous NO generation and that of NO on MT biosynthesis. To analyze the interaction between MT and NO, seedlings with three leaves were pretreated with 100 μM MT, 75 μM SNP, 100 μM 2-(4-carboxyphenyl)-4,4,5,5-tetramethylimidazoline-1-oxyl-3-oxide (cPTIO, a specific scavenger of NO) +100 μM MT, 50 μM p-chlorophenylalanine (p-CPA, MT synthesis inhibitor) +75 μM SNP, or deionized water (control). At 24 h after cold stress, the pretreated seedlings were subjected to 5°C in growth chambers. At 24 h after chilling treatment, the gas exchange parameter, fluorescence parameters, and the key enzymes in the Calvin–Benson cycle were detected. At 48 h after cold stress, the accumulation of ROS and gene expression and the activity of antioxidant enzymes were measured. The deionized water treatment under cold stress was considered as H_2_O treatment to distinguish from the control at normal temperature conditions.

### Measurement of MT and Activity of Its Synthase Enzymes

Endogenous MT was detected by using high-performance liquid chromatography–triple quadrupole mass spectrometry (HPLC–MS, Thermo Fisher Scientific, TSQ Quantum Access, Waltham, MA, USA) according to the method of Bian et al. (2018). Briefly, freeze-dried leaves (0.1 g) were ground in 9 ml 100% methanol, extracted with cryogenic ultrasound for 15 min, and vortexed in darkness at 4°C for 24 h. The homogenates were centrifuged at 10,000 × g for 15 min at 4°C, and the supernatants were dried with nitrogen gas. The residues were dissolved in 3 ml 5% methanol, centrifugated at 10,000 × g for 15 min to remove pigment and impurities, and then purified further with a C_18_ solid-phase extraction column (Bond Elut C_18_, 100 mg 1 ml, 100/pk, Agilent Technologies, Inc., Folsom, Santa Clara, CA, USA). The residue was dissolved in 1 ml 90% chromatographic methanol (containing 0.05% acetic acid), and the filtrate was used for the HPLC-MS analysis. The samples were separated by using a Hypersil Gold C_18_ column (Thermo Fisher Scientific, Waltham, MA, USA, 100 ×2.1 mm, 1.9 μm) at a flow rate of 0.3 ml·min^−1^ and a column temperature of 25°C. About 90% chromatographic methanol and 0.05% acetic acid were used as mobile phases. The separated components were further quantitatively analyzed by MS using triple quadrupole MS with electrospray ionization (ESI) in the positive mode. The MT and 2-hydroxymelatonin in cucumber leaves were determined by an external standard method and were calculated according to the peak area.

The activities of tryptophan decarboxylase (TDC) and acetylserotonin O-methyltransferase (ASMT) were measured by the ELISA method using ELISA kits for TDC and ASMT in plants (SU-B91337 and SU-B91345, Quanzhou Kenuodi Biotechnology Co., Ltd., Quanzhou, China), respectively.

### Determination of Chlorophyll Fluorescences

After seedlings were dark adapted for 45 min, the maximum photochemical efficiency of photosystem II (PSII) (*F*_v_/*F*_m_) and the actual photochemical efficiency of PSII (*Φ*_PSII_) were detected using an Imaging-PAM Chlorophyll Fluorometer (IMAGING-PAM, Walz, Wurzburg, Germany). *F*_v_/*F*_m_ and *Φ*_PSII_ were calculated as follows: *F*_v_/*F*_m =_ (*F*_m_-*F*_0_)/*F*_m_; *Φ*_PSII =_ (*F*_m_'–*F*_s_)/*F*_m_' (Demmig-Adams and Adams, 1996). The initial fluorescence (*F*_0_) was estimated after turning on the measuring beam, followed by a 0.8-s saturation pulse (3,000 μmol m^−2^·s^−1^) to obtain maximum fluorescence (*F*_m_). Steady-state fluorescence (*F*_s_) was recorded using the actinic light intensity of 400 μmol m^−2^·s^−1^ for 5 min. The chlorophyll fluorescence imaging of cucumber leaves was visualized using the method of Tian et al. (2017), with a variable chlorophyll fluorescence imaging system (Imaging PAM, Walz, Wurzburg, Germany), which consists of a CCD camera, LED lights, and a controlling unit connected to a PC running a dedicated software (Imaging Win 2.3, Walz, Wurzburg, Germany).

The dark-adapted leaves were used to determine the transient chlorophyll fluorescence and 820 nm reflection with an integral multifunctional plant efficiency analyzer (M-PEA, Hansatech, King's Lynn, Norfolk, UK) as described by Liu et al. (2020). The parameters were calculated as follows (Chen et al., 2016): relatively variable fluorescence at time *t* (*V*_t_) = (*F*_t_ – F_0_)/(*F*_m_ – *F*_0_), Δ*V*_t_ = *V*_t_ – *V*_t(control)_; the performance index on the absorption basis (PI_ABS_) = RC/ABS × [ϕ_P0_/(1 – ϕ_P0_)] × [ψ_0_/(1 – ψ_0_)]; the efficiency of an electron moving beyond *Q*_A_ (ψ_0_) = ET/TR =1 – *V*_J_; and the quantum for heat dissipation (ϕ_D0_) = *F*_0_/*F*_m_. The amplitude of the 820 nm reflection is (Δ*I*/*I*_0_) = (*I*_0_ – *I*_m_)/*I*_0_ (Salvatori et al., 2014), where *I*_0_ is the initial reflection signal between 0.4 and 10 ms and *I*_m_ is the minimum reflection signal under 820 nm far-red illumination (Zhang et al., 2011).

### Measurement of MDA Content, EL, and Chilling Injury Index

Malondialdehyde content was determined using the TBA colorimetric method as described by Dong et al. (2013). EL was measured using the method of Dong et al. (2013). A total of 0.3 g of each leaf sample was incubated in a tube with 20 ml of deionized water at 25°C, and electrical conductivity (EC) of the bathing solution was estimated at 3 h, which was named EC1, using a conductivity meter (DDB-303A, Shanghai Precision Scientific Instrument Co., Ltd., Shanghai, China). Then, the samples were boiled for 30 min, and EC of the bathing solution was measured after cooling to room temperature, and named EC2. EL was calculated as follows: EL = EC1/EC2 ×100. The stressed seedlings were graded according to Semeniuk et al. (1986), and the chilling injury index (CI) was calculated as follows: CI = Σ (plants of different grade × grade)/[total plants ×5 (the maximum grade)].

### NO Content and Nitrate Reductase Activity Assay

Nitric oxide was extracted from the second apical leaves and quantified using the method specified in the NO kit (Nanjing Jiancheng Bioengineering Institute, Nanjing, China). The intracellular NO was visualized using the NO fluorescent probe diaminofluorescein-FM diacetate (DAF-FM DA; Beyotime, Shanghai, China) for localization according to the instructions. Fluorescent was formed in the presence of NO (excitation at 495 nm and emission at 515 nm) and visualized using an inverted fluorescence microscope (Leica DMi8; Leica, Wetzlar, Germany). Nitrate reductase (NR) activity was detected using the method of Zhao et al. (2002).

### Gas Exchange Parameters Assay

The photosynthetic rate (*P*_n_) for the second apical leaves was detected using a portable photosynthetic system (Ciras-3, PP-systems International, Hitchin, Hertfordshire, UK). The determination conditions were maintained as follows: 600 μmol m^−2^·s^−1^ PFD, 380 mg·L^−1^ CO_2_ concentration, and 25 ± 1°C leaf temperature. Following the method of Ethier and Livingston (2004), the light-saturated photosynthetic rate (Asat) was detected.

### Quantitative and Histochemical Detection of ROS

Hydrogen peroxide (H_2_O_2_) was extracted from 0.5-g leaf samples and quantified according to the instructions specified in the H_2_O_2_ kit (A064-1, Nanjing Jiancheng Bioengineering Institute, Nanjing, China). The superoxide anion (O_2_^−^) content was measured as described by Wang and Luo (1990). Cellular H_2_O_2_ and O_2_^−^ were visualized at the subcellular level using the H_2_O_2_ fluorescent probe 2′, 7′-dichlorodihydrofluorescein diacetate (H_2_DCFDA) (MCE, Cat. No. HY-D0940, Shanghai, China) and dihydroethidium (DHE) (Fluorescence Biotechnology Co., Ltd., Cat. No. 15200, Beijing, China), respectively, using the method of Zhang et al. (2020).

### Measurement of Antioxidant Enzyme Activity

Fresh samples (0.5 g) were ground in 5 ml of 50 mM ice-chilled phosphate buffer solution (pH 7.8) containing 0.2 mM EDTA and 1% (w/v) PVP. The homogenates were centrifuged at 12,000 × g for 20 min at 4°C, and the supernatant was used to determine the activity of antioxidant enzymes. Superoxide dismutase (SOD) activity was measured using the method of Stewart and Bewley (1980). Peroxidase (POD) activity was determined following the method of Omran (1980) and was quantified using the absorbance changes at 470 nm over 1 min. Ascorbate peroxidase (APX) activity was determined as described by Nakano and Asada (1987) and was determined by the changes of absorbance at 290 nm over 1 min. Glutathione reductase (GR) activity was assayed based on the method of Foyer and Halliwell (1976) and was expressed as the decreasing rate in the absorbance of NADPH at 340 nm over 1 min.

### Glutathione and Ascorbic Acid Content Assays

We measured the contents of reduced glutathione (GSH) and oxidized glutathione (GSSG) using a GSH content kit (GSH-2-W, and GSSG-2-W, Suzhou Keming Biotechnology Co., Ltd., Suzhou, China) according to the instructions. The contents of ascorbic acid (AsA) and dehydroascorbic acid (DHA) were estimated using the method of Law et al. (1983).

### Activity of the Key Enzymes in Calvin–Benson Cycle

Ribulose-1,5-bisphosphate carboxylase (RuBPCase) activity was assayed spectrophotometrically using the RuBPCase kit (RUBPS-2A-Y, Suzhou Keming Biotechnology Co., Ltd., Suzhou, China) according to the instructions. Rubisco activase (RCA) activity was detected by ELISA with an RCA ELISA kit (SU-B91104, Quanzhou Kenuodi Biotechnology Co., Ltd., Quanzhou, China).

### RNA Extraction and Gene Expression Analysis

Total RNA was extracted using TransZol reagent (Transgen, Beijing, China) and reverse transcribed with the HiScript® III RT SuperMix for qPCR (+gDNA wiper) (Vazyme, Nanjing, China). Quantitative real-time PCR (qRT-PCR) for *TDC, T5H, SNAT, ASMT, NR*, antioxidant genes, key genes of the Calvin–Benson cycle, and cold stress-responsive genes in cucumber seedlings were analyzed using ChamQ™ Universal SYBR® qPCR MasterMix (Vazyme, Nanjing, China) according to the instructions. We used the cucumber β-actin gene (Gene ID: Solyc11g005330) as a constitutively expressed internal control. The synthesis of primers by BGItech is shown in Table 1.

**Table 1:** The primer sequences of quantitative real-time PCR (qRT-PCR).

**Primer names**	**Sequences(5' → 3')**
ß*-Actin*	F: CCACGAAACTACTTACAACTCCATC
	R: GGGCTGTGATTTCCTTGCTC
*TDC*	F: ATAAATGGTTCTTCTCGGCGCCAG
	R: GTTAATCATATTCGACTTCTGGT
*T5H*	F: AGCTTGTGCAGGCTACCAACT
	R: GAACGTTGGAACAAACTTGTG
*SNAT*	F: AGTCCCCTGTTTCAGAGGAGAAT
	R: AGATTCCGATAAAACTCTACCAC
*ASMT*	F: ATTGGAAGTTTAGTTGATGTGGGA
	R: AGCATCAGCCTTGGGAATGGAAT
*NR*	F: CAAGAAAGAGCTGGCTATGG
	R: CTACATGGGATGGCAAGACT
*SOD*	F: GGAAAGATGTGAAGGCTGTGG
	R: GCACCATGTTGTTTTCCAGCAG
*POD*	F: GGTTTCTATGCCAAAAGCTGCCC
	R: CAGCTTGGTTGTTTGAGGTGGAG
*APX*	F: GTGCTACCCTGTTGTGAGTG
	R: AACAGCGATGTCAAGGCCAT
*GR*	F: TGATGAGGCTTTGAGTTTAGAGGAG R: AACTTTGGCACCCATACCATTC
*rbcL*	F: GCTATGGAATCGAGCCTGTTG
	R: CCAAATACATTACCCACAATGGAAG
*RCA*	F: AAAGTGGGCTGTAGGCGTTG
	R: CTTTTCTATTGTCATCTTCGGTTGG
*ICE1*	F: CGCATCGAGTTGGCTCTGGTG
	R: GTCCTCATCGCCGTTCATCTTCC
*CBF1*	F: TACAGAGGAGTCAGGAGGA
	R: AGAATCGGCGAAATTGA
*COR47*	F: CACTTTGAGAGGACATTTGATG
	R: AGAAGCTCCAATTTTGACTTG

### SDS-PAGE and Immunoblot Analysis

Fresh leaves were ground in liquid nitrogen and homogenized in extraction buffer (20 mM tricine, 1 mM sodium ascorbate, 400 mM sorbic alcohol, 10 mM NaHCO_3_, 5 mM EDTA·Na_2_, and 5 mM MgCl_2_) for protein extraction and then centrifugated at 2,000 × g for 15 min. After adjusting to the same concentration, the proteins were mixed with 5 × loading buffer (CW0027S, Beijing ComWin Biotech Co., Ltd., Beijing, China) and boiled at 100°C for 15 min. The proteins were separated using 10% sodium dodecyl sulfate-polyacrylamide gradient gel electrophoresis (SDS-PAGE). For D1, Photosystem I reaction center subunit II (PsaD), RbcL, and RCA detection, antibodies specific to the D1, PsaD, RbcL, and RCA proteins (ATCG00020, AT1G03130, ATCG00490, AT2G39730, PhytoAB Company, San Francisco, CA, USA) were used, followed by incubation with horseradish POD-conjugated anti-rabbit IgG antibody (ComWin Biotech Co., Ltd., Beijing, China). For C-repeat-binding factor (CBF1) detection, an antibody specific to CBF1 proteins (GenScript Co, Nanjing, China) was used, followed by incubation with a horseradish POD-conjugated anti-rabbit IgG antibody (ComWin Biotech Co., Ltd., Beijing, China). The immunoreaction was detected with an eECL Western Blot Kit (CW00495, ComWin Biotech Co., Ltd., Beijing, China). We recorded chemiluminescence on blots using the ChemiDoc™ XRS imaging system (Bio-Rad Laboratories, Inc., Hercules, CA, USA).

### Statistical Analysis

All experiments were performed at least in triplicate, and the results are shown as mean ± one SD. All data were analyzed statistically using DPS software. Statistical analysis of the values was performed by Duncan's multiple range test (DMRT), and comparisons with *p* < 0.05 were considered significantly different.

## Results

### The Response of Endogenous MT to Cold Stress and Its Relationship With Cold Tolerance in Cucumber

**Figure 1:**
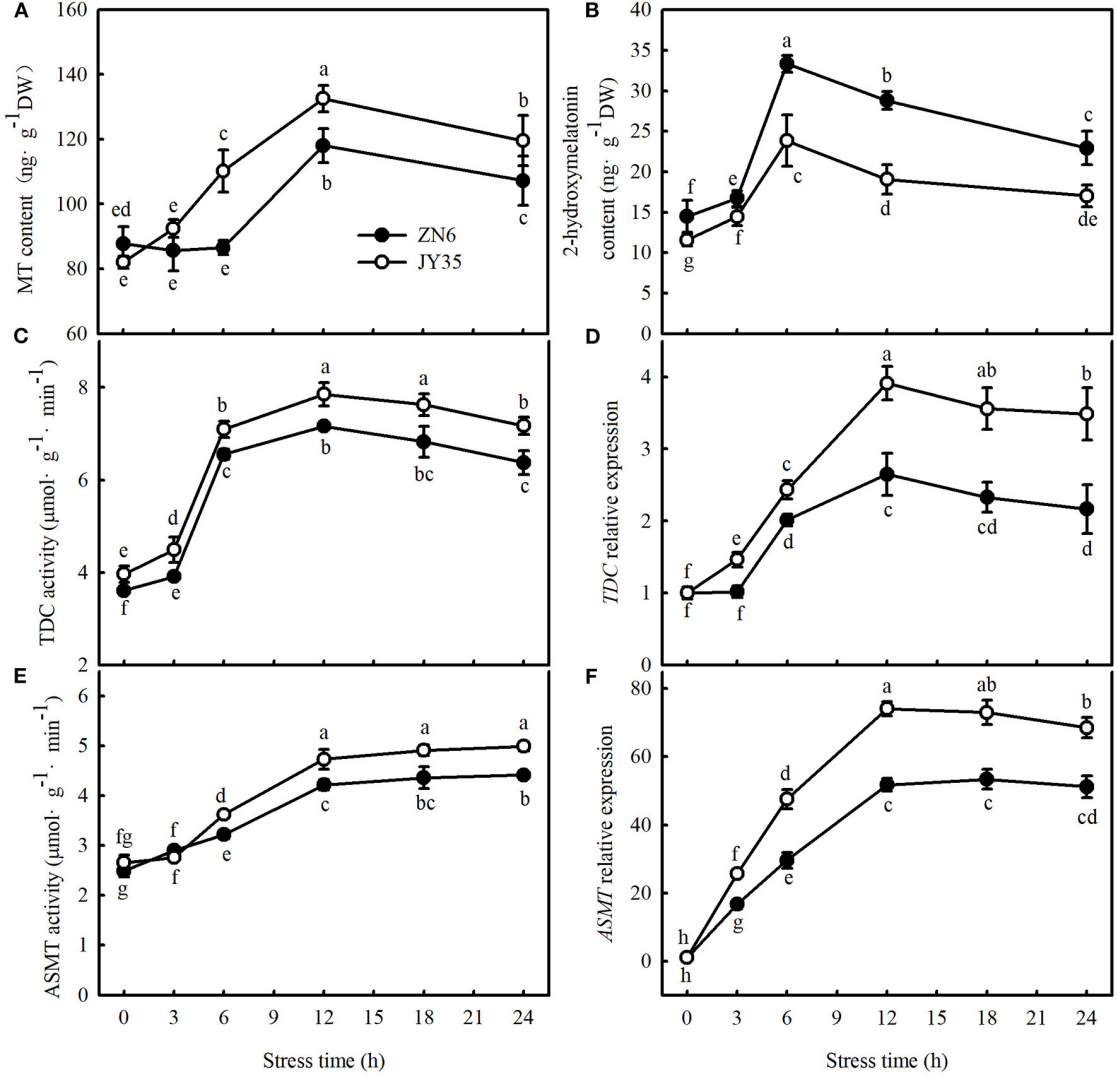
Response of endogenous melatonin (MT) to cold stress in cucumber seedlings. **(A)** MT content; **(B)** 2-hydroxymelatonin content; **(C)** tryptophan decarboxylase (TDC) activity; **(D)** relative messenger RNA (mRNA) expression of *TDC*; **(E)** acetylserotonin O-methyltransferase (ASMT) activity; and **(F)** relative mRNA expression of *ASMT*. Seedlings were treated at 5°C for 24 h and sampled every 3 h. Data are the means ± SD (*n* = 3). Different letters indicate a significant difference between samples according to Duncan's new multiple range test (*p* < 0.05).

We first determined whether endogenous MT levels are related to cold tolerance by comparing the changes in endogenous MT, the activity and relative mRNA expression of MT synthase enzymes in response to cold stress, and the *F*_v_/*F*_m_, *Φ*_PSII_, MDA content, EL, and CI in different varieties of cucumber. As shown in Figure 1, cold stress induced greater activities and relative mRNA expression of *TDC* and *ASMT* (key enzymes of MT biosynthesis) and consequently promoted endogenous MT and its metabolite accumulation, 2-hydroxymelatonin, in cucumber seedlings. The increases in MT content and the activities and relative mRNA expression of *TDC* and *ASMT* were most significant after 12 h at 5°C, but an increase in 2-hydroxymelatonin was most significant after the exposure of seedlings to cold stress for 6 h. Compared to the “ZN6” seedlings, the “JY35” seedlings exhibited significantly higher activities and relative mRNA expressions of *TDC* and *ASMT*, as well as MT content (*p* < 0.05), but much lower 2-hydroxymelatonin accumulation (*p* < 0.05) following cold stress.

**Figure 2:**
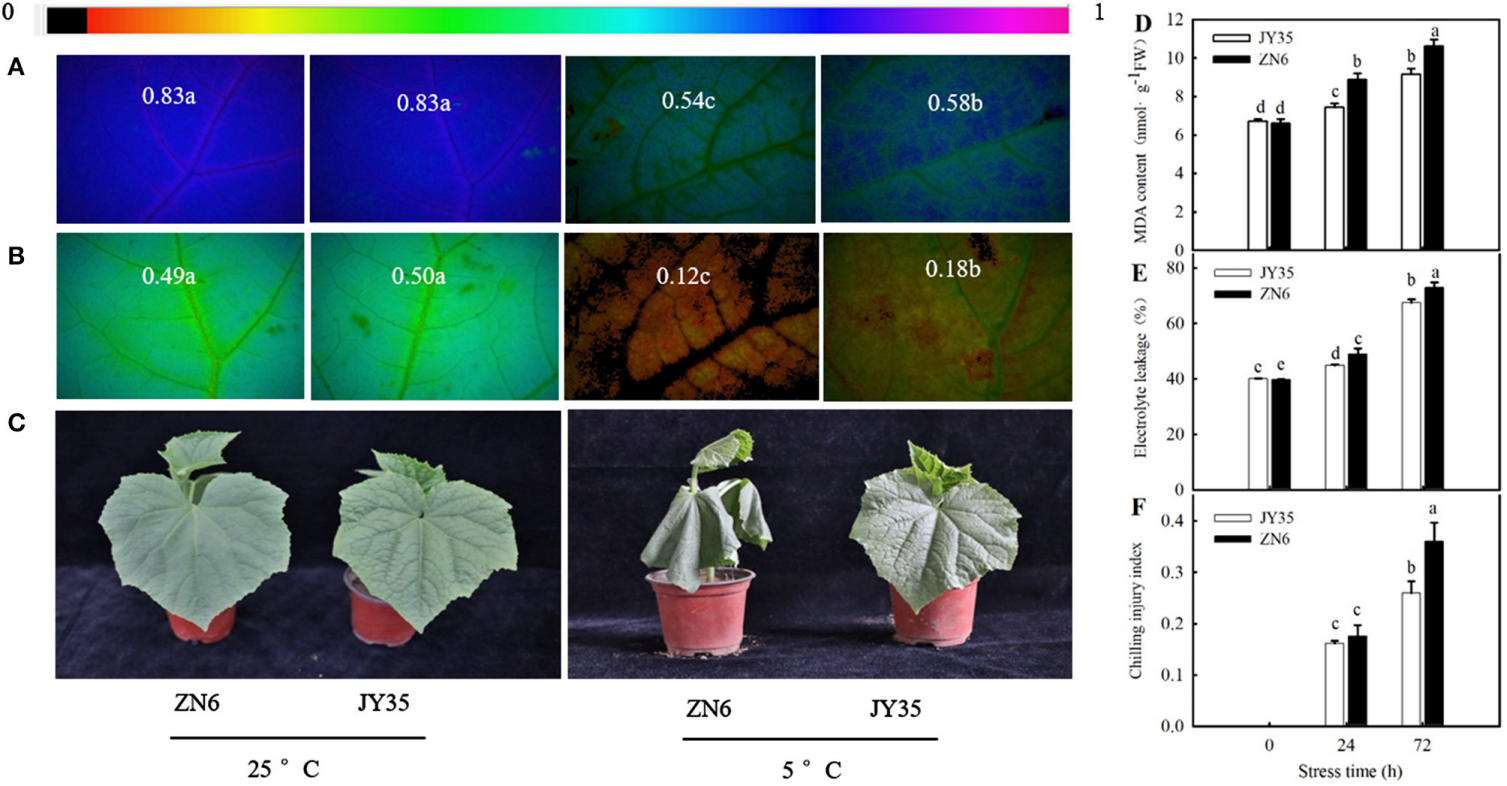
Comparison of cold tolerance in the two different cucumber varieties. Images of **(A)** *F*_v_/*F*_m_ and **(B)** *Φ*_PSII_, respectively. The false color code depicted at the top of the image ranging from 0 (black) to 1 (purple) represents the degree of photoinhibition at photosystem II (PSII). **(C)** Seedling phenotype. Three-leaf stage seedlings were treated at 5°C for 24 h; **(D)** Malondialdehyde (MDA) accumulation, **(E)** Electrolyte leakage (EL) rate, and **(F)** Chilling injury index (CI), respectively. Seedlings were exposed to 5°C for 72 h. Data are represented as mean ± SD (*n* = 3). Different letters indicate a significant difference between samples according to Duncan's new multiple range test (*p* < 0.05).

Cold stress caused a remarkable decrease (*p* < 0.05) in *F*_v_/*F*_m_ and *Φ*_PSII_ and an increase in MDA content, EL, and CI. The decreases in *F*_v_/*F*_m_ and *Φ*_PSII_ and increases in MDA content, EL, and CI in the “JY35” seedlings were significantly lower than those in the “ZN6” seedlings (Figures 2A,B,D–F). Histochemical observation using an inverted fluorescence microscope for *F*_v_/*F*_m_ and *Φ*_PSII_ (Figures 2A,B) agreed with the results determined by a chlorophyll fluorometer. Figure 2C shows that cold stress resulted in visible damages such as wilting and necrosis in cucumber leaves. The “JY35” seedlings exhibited minor damage compared to the “ZN6” seedlings, as evidenced by the unaided visual observations. These data indicated that MT is induced by cold stress and is involved in the response of cucumber seedlings to cold stress. Amplitude variation of endogenous MT in different varieties is positively correlated with cold tolerance.

### MT Improves Cold Tolerance and NO Accumulation in Cucumber Seedlings

**Figure 3:**
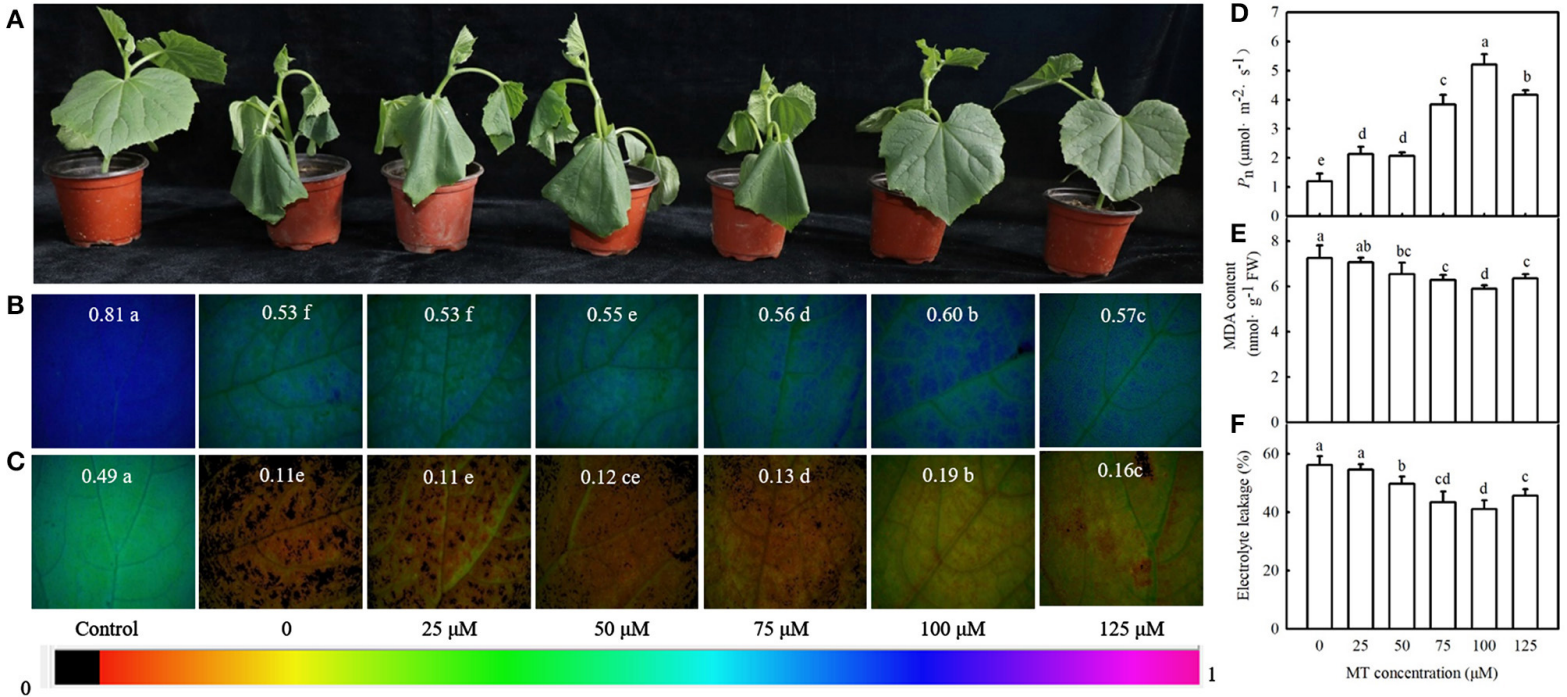
Effects of MT on cold tolerance of cucumber seedlings under cold stress. **(A)** Seedling phenotype; **(B)** images of *F*_v_/*F*_m_, **(C)** *Φ*_PSII_; **(D)** *P*_n_. Three-leaf stage seedlings were foliar sprayed with MT (0, 25, 50, 75, 100, or 125 μM) for 24 h and then were exposed to 5°C for 24 h. **(E)** MDA content; **(F)** EL rate. Seedlings were foliar sprayed with MT (0, 25, 50, 75, 100, or 125 μM) for 24 h and then were exposed to 5°C for 72 h. Data are represented as mean ± SD (*n* = 3). Different letters indicate a significant difference between samples according to Duncan's new multiple range test (*p* < 0.05).

We then examined the effect of different concentrations of MT on the seedling symptoms, *F*_v_/*F*_m_, *Φ*_PSII_, *P*_n_, MDA content, and EL under cold stress. Figure 3A reveals that exogenous MT alleviated the foliar damage in cucumber seedlings caused by cold stress, and 100 μM treatment was most noticeable. MT also increased *F*_v_/*F*_m_, *Φ*_PSII_, and *P*_n_ but decreased MDA accumulation and EL. This increase in *F*_v_/*F*_m_, *Φ*_PSII_, and *P*_n_ or a decrease in MDA and EL in seedlings was enhanced at a low concentration of MT but was inhibited when MT concentration exceeded 100 μM (Figures 3B–F). The seedlings treated with 100 μM MT displayed significantly higher *F*_v_/*F*_m_, *Φ*_PSII_, and *P*_n_ but much lower MDA and EL than other treated seedlings (*p* < 0.05). These data indicate that MT improves the cold tolerance of cucumber in a concentration-dependent manner. Hence, 100 μM MT was used in subsequent experiments.

**Figure 4:**
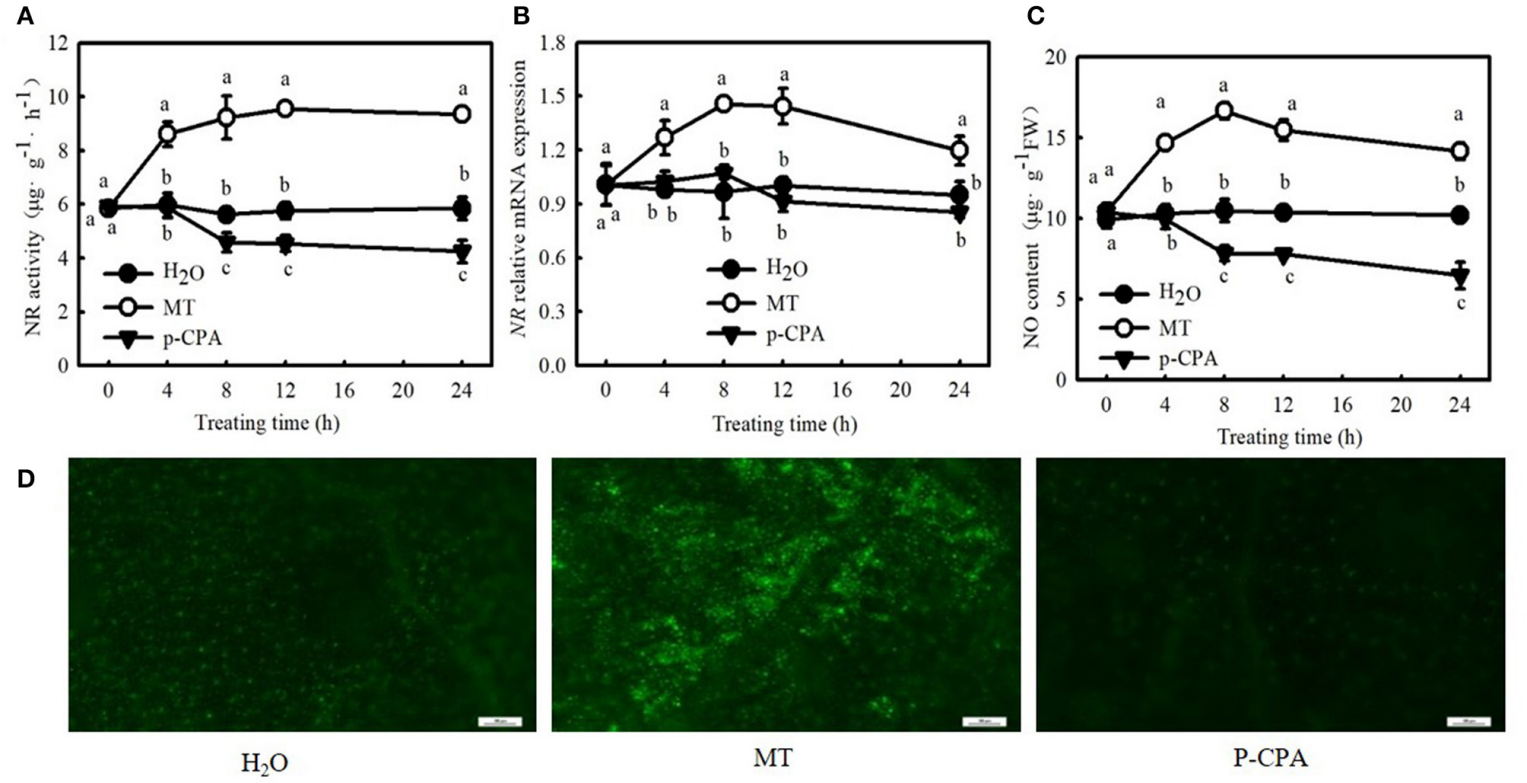
Effects of MT on the nitrate reductase (NR) activity, *NR-*relative mRNA expression, and nitric oxide (NO) content in cucumber seedlings under normal temperature. **(A)** NR activity; **(B)** *NR-*relative mRNA expression; and **(C)** NO content. Three-leaf stage seedlings were foliar sprayed with 100 μM MT, 50 μM p-CAP (a biosynthetic inhibitor of MT), or distilled water (control) for 24 h. **(D)** NO inverted fluorescence microscope imaging at 25°C for 8 h. Data are represented as mean ± SD (*n* = 3). Different letters indicate a significant difference between samples according to Duncan's new multiple range test (*p* < 0.05).

**Figure 5:**
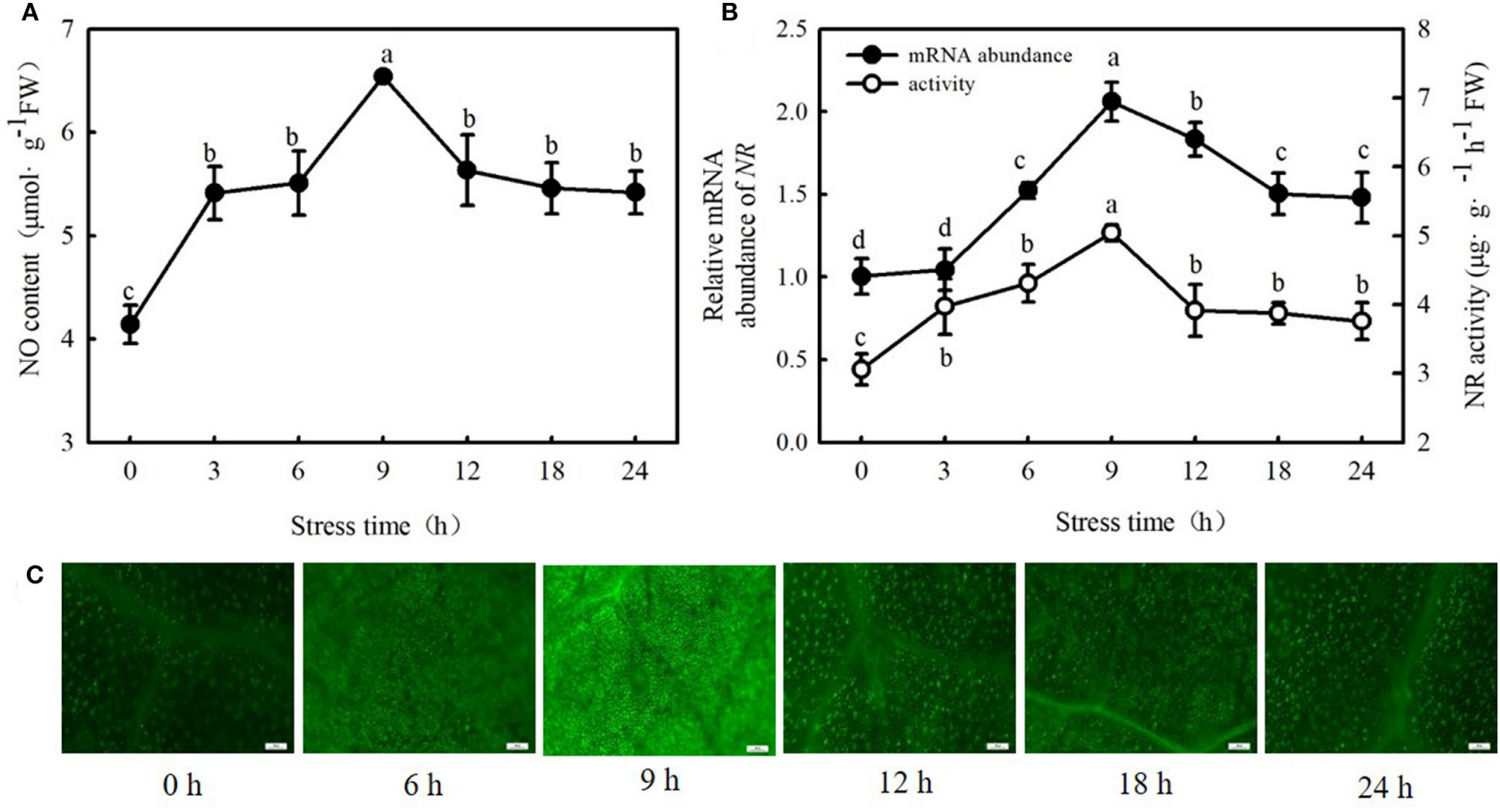
Response of NO, activities, and relative mRNA expressions of NR to cold stress in cucumber seedlings. **(A)** NO content; **(B)** NR activity and *NR-*relative mRNA expression; and **(C)** NO inverted fluorescence microscope imaging. Seedlings were treated at 5°C for 24 h, and sampled every 3 h. Data are represented as mean ± SD (*n* = 3). Different letters indicate a significant difference between samples according to Duncan's new multiple range test (*p* < 0.05).

**Figure 6:**
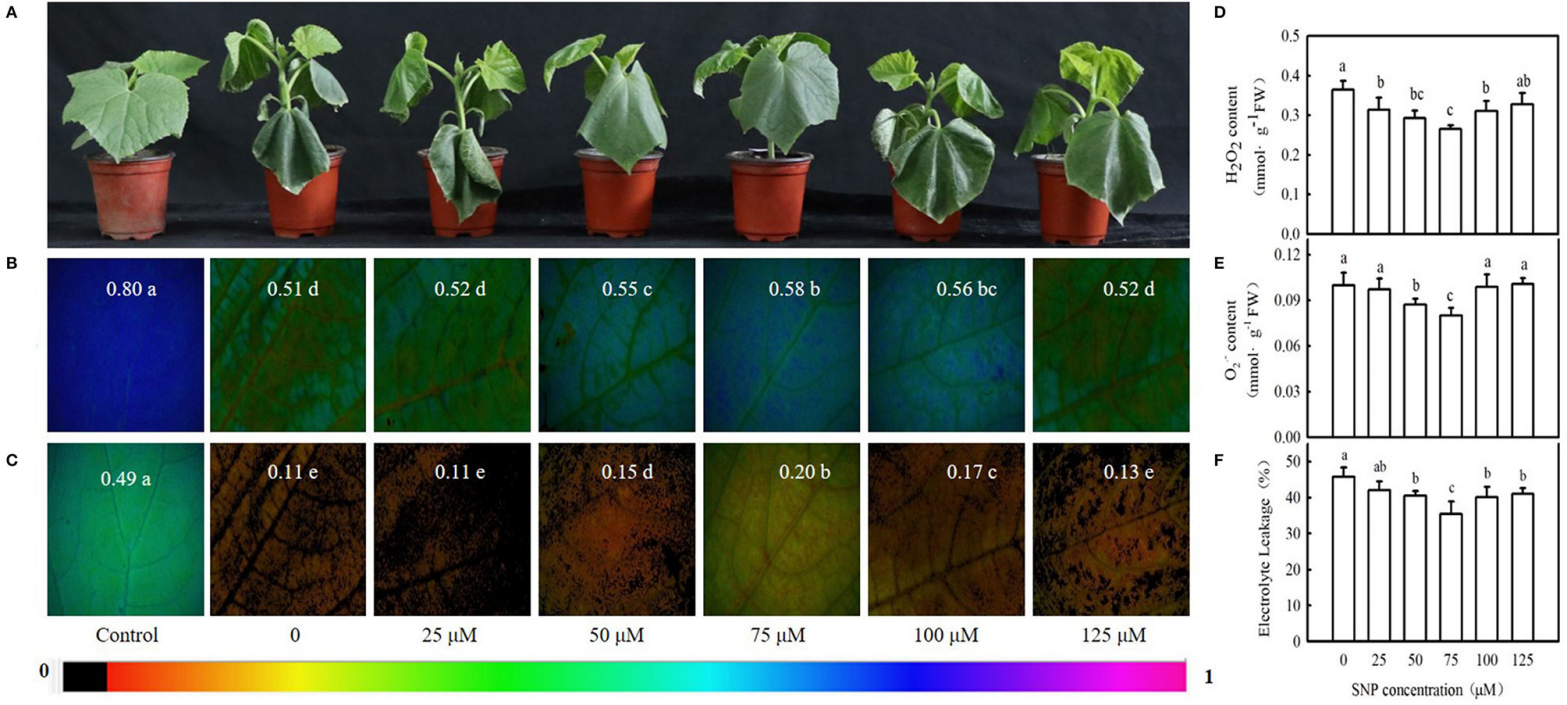
Effect of sodium nitroprusside (SNP) on cold tolerance of cucumber seedlings. **(A)** Seedling phenotype, **(B,C)** Images of *F*_v_/*F*_m_ and *Φ*_PSII_, respectively. Three-leaf stage seedlings were foliar sprayed with SNP (0, 25, 50, 75, 100, or 125 μM, respectively) for 24 h, and then were exposed to 5°C for 24 h. The false color code depicted at the top of the image ranging from 0 (black) to 1 (purple) represents the degree of photoinhibition at PSII. **(D)** Hydrogen peroxide (H_2_O_2_) content; **(E)** O_2_^−^ content; and **(F)** EL rate. Seedlings were foliar sprayed with SNP (0, 25, 50, 75, 100, or 125 μM, respectively) for 24 h and then were exposed to 5°C for 72 h. Data are represented as mean ± SD (*n* = 3). Different letters indicate a significant difference between samples according to Duncan's new multiple range test (*p* < 0.05).

Interestingly, 100 μM MT significantly increased NO content, NR (a key enzyme for NO biosynthesis) activity, and *NR*-relative mRNA expression in cucumber seedlings (Figures 4A–C; *p* < 0.05). However, p-CPA-treated seedlings showed lower or similar NO content, NR activity, and relative mRNA expression compared with H_2_O-treated seedlings. The NO accumulation observed by inverted fluorescence microscope (Figure 4D) was consistent with the results of biochemical analysis. This finding implies that NO may play a crucial role in MT-induced cold tolerance of cucumber seedlings. To verify this inference, we measured the response of endogenous NO to cold stress, and the effect of exogenous NO on chilling injury symptoms, *F*_v_/*F*_m_, *Φ*_PSII_, ROS accumulation, and EL in cucumber seedlings. We found that NR activity, *NR*-relative mRNA expression, and NO accumulation in cucumber seedlings also increased under cold stress, and the highest values were reached after 9 h of cold stress (Figure 5). Importantly, SNP (NO specific donor) increased *F*_v_/*F*_m_ and *Φ*_PSII_ while decreasing MDA accumulation and EL in cucumber seedlings, confirming that endogenous NO also enhances the cold tolerance of cucumber seedlings. Seedlings treated with 75 μM SNP exhibited the highest *F*_v_/*F*_m_ and *Φ*_PSII_ but the lowest ROS accumulation and EL among the six SNP treatments (Figure 6), so 75 μM SNP was used in subsequent experiments.

**Figure 7:**
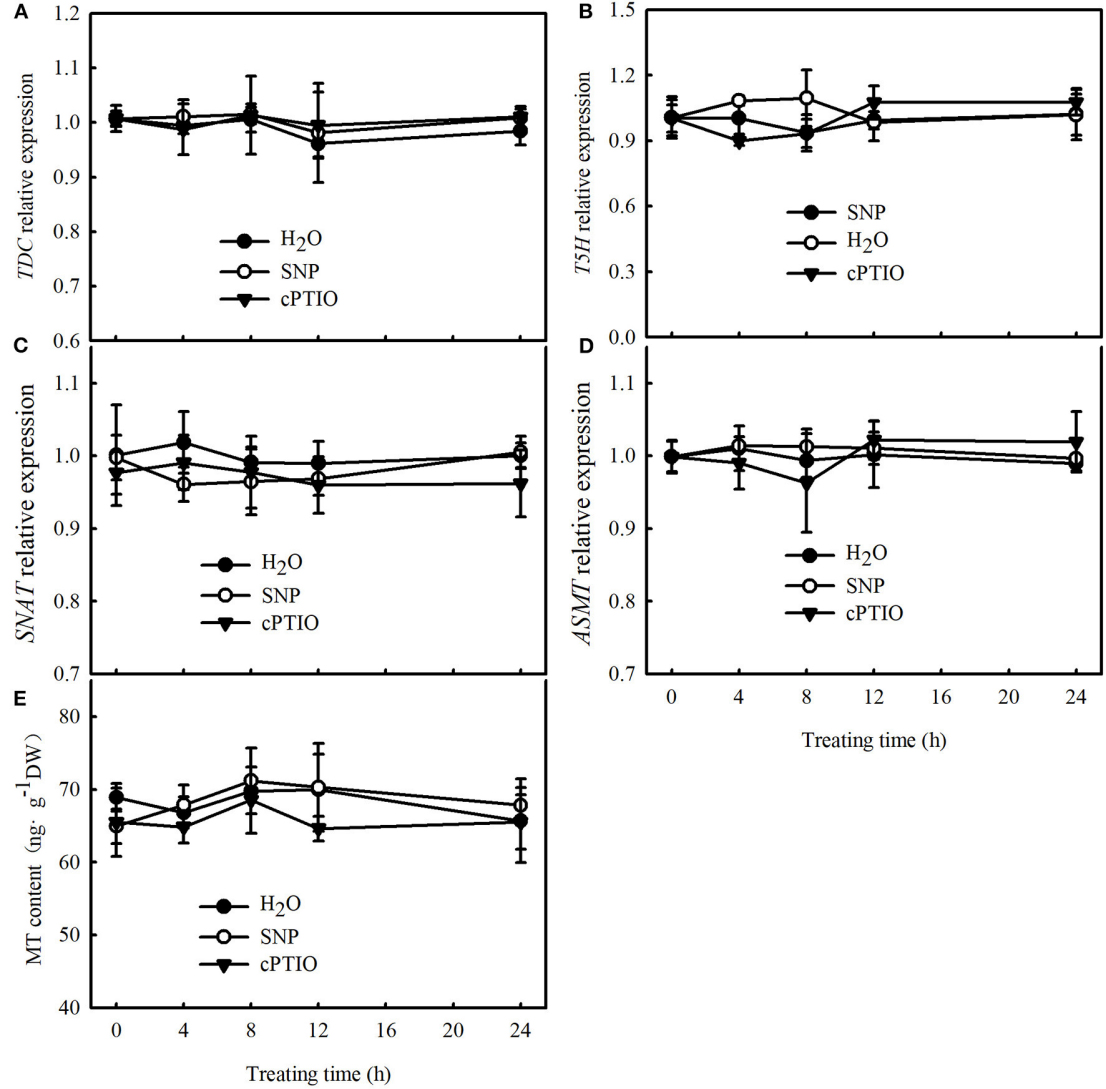
Effects of SNP on MT content and key genes of MT biosynthesis, relative mRNA expression in cucumber seedlings. **(A–D)** Relative mRNA expression of *TDC, T5H, SNAT*, and *ASMT*, respectively; **(E)** MT content. Three-leaf stage seedlings were foliar sprayed with 75 μM SNP, 100 μM 2-(4-carboxyphenyl)-4,4,5,5-tetramethylimidazoline-1-oxyl-3-oxide (cPTIO) (a NO biosynthetic inhibitor), or distilled water (control) for 24 h. Data are represented as mean ± SD (*n* = 3).

To further explore the upstream and downstream relationship of MT and NO signals under cold stress, we determined the effect of SNP on the mRNA abundance of key genes of MT biosynthesis and MT content in cucumber seedlings at normal temperature. No differences were observed in the relative mRNA expression of *TDC, T5H, SNAT*, and *ASMT* and in the MT content between SNP, cPTIO, or control treatments (Figure 7). These results indicate that NO may function as a downstream signal for MT-induced cold tolerance in cucumber seedlings.

### Exogenous MT and SNP Increased ROS Scavenging Activity in Cucumber Seedlings Under Cold Stress

**Figure 8:**
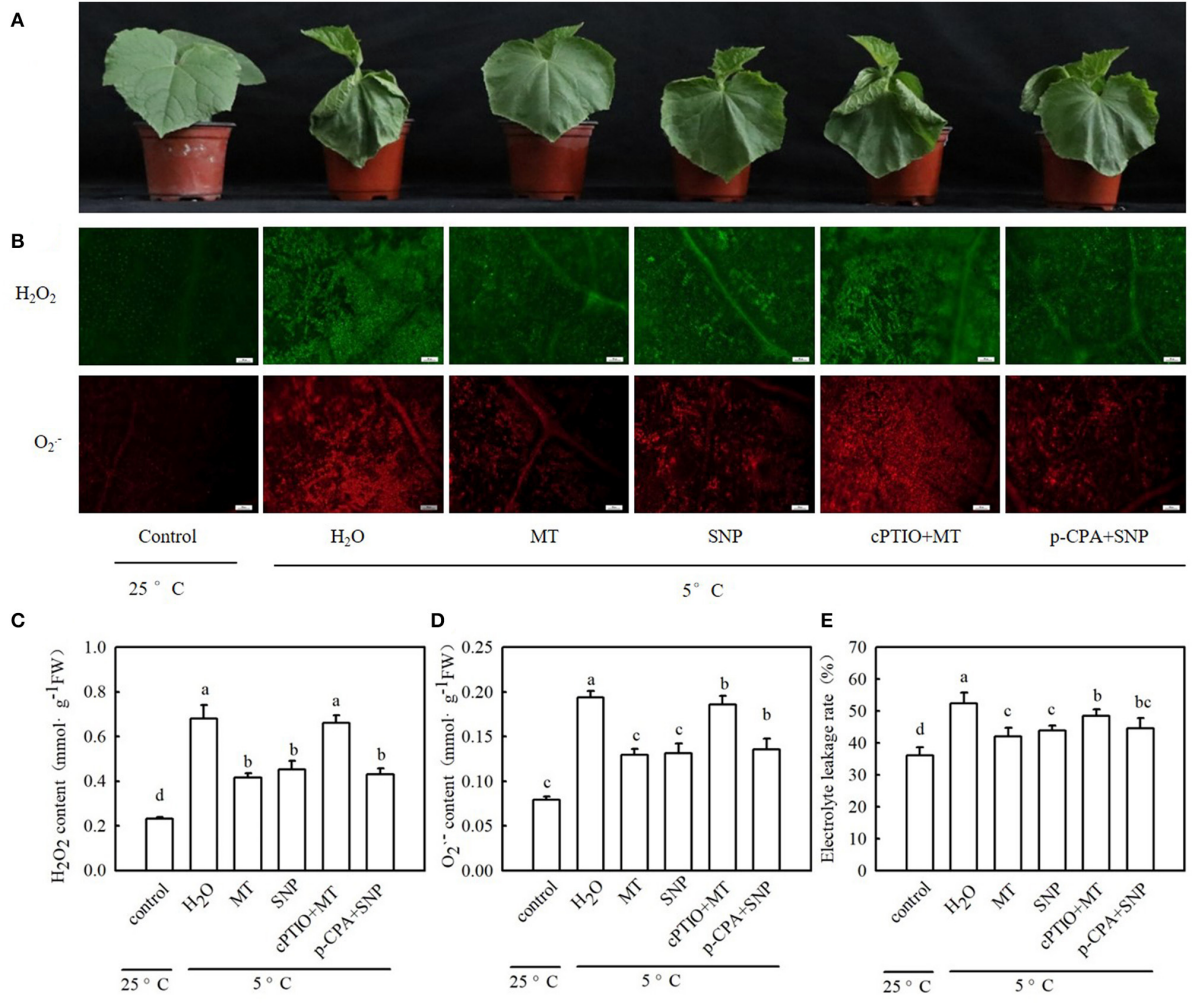
Interactive effects of MT and SNP on the reactive oxygen species (ROS) accumulation and EL in cucumber seedlings under cold stress. **(A)** Seedling phenotype; **(B)** Inverted microscope imaging of H_2_O_2_ and O_2_^−^; **(C)** H_2_O_2_ content; **(D)** O_2_^−^ content; and **(E)** EL. Three-leaf stage seedlings were foliar sprayed with 100 μM MT, 75 μM SNP, 100 μM cPTIO+100 μM MT, 50 μM p-chlorophenylalanine (p-CPA) +75 μM SNP, or deionized water (control) for 24 h and then were exposed to 5°C for 48 h. Data are represented as mean ± SD (*n* = 3). Different letters indicate a significant difference between samples according to Duncan's new multiple range test (*p* < 0.05).

**Figure 9:**
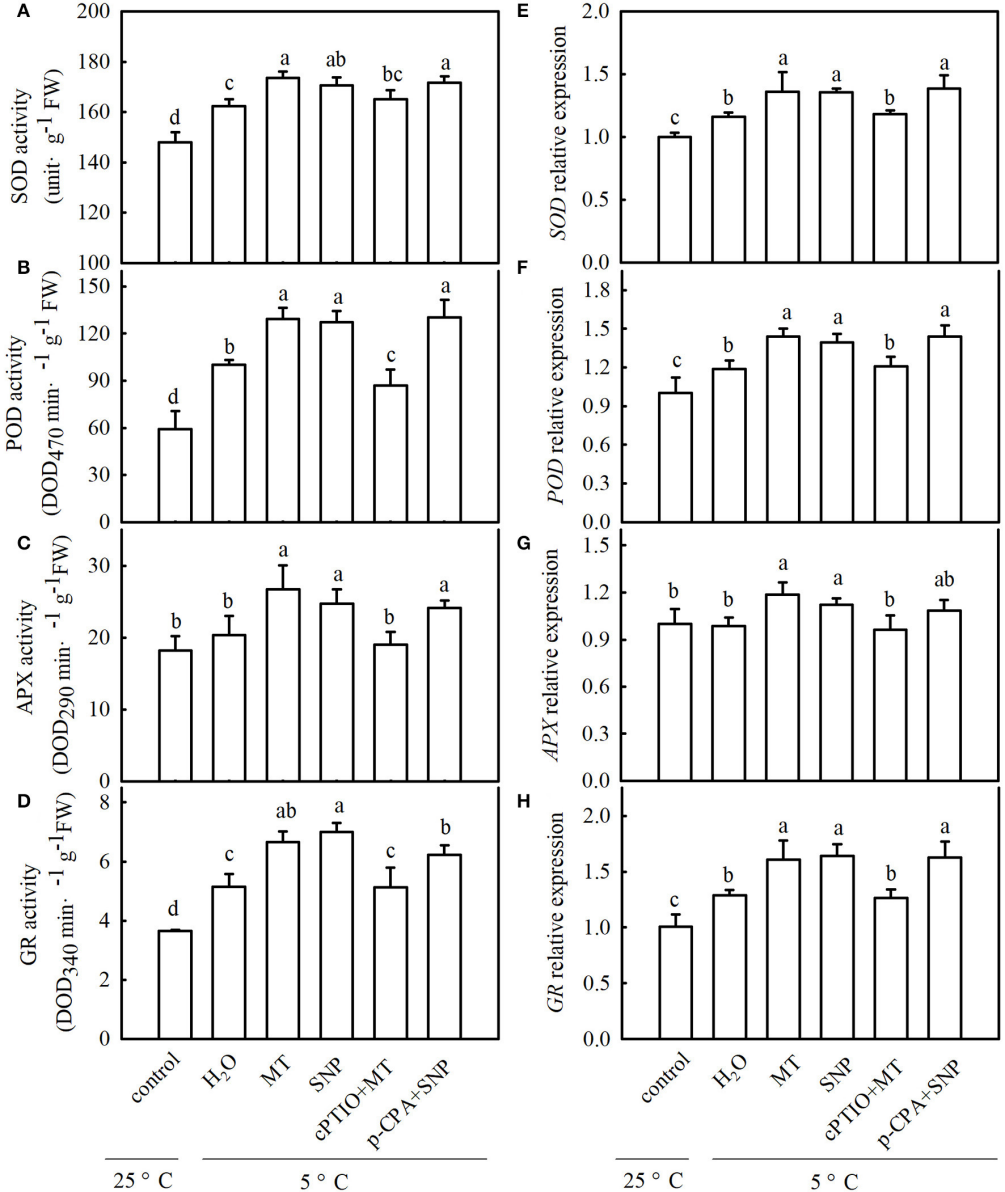
Interactive effects of MT and SNP on activities and relative mRNA expression of antioxidant enzymes in cucumber seedlings under cold stress. **(A–D)** Activities of superoxide dismutase (SOD), peroxidase (POD), ascorbate peroxidase (APX), and glutathione reductase (GR), respectively. **(E–H)** Relative mRNA expression of *SOD, POD, APX*, and *GR*, respectively. Three-leaf stage seedlings were foliar sprayed with 100 μM MT, 75 μM SNP, 100 μM cPTIO+100 μM MT, 50 μM p-CPA + 75 μM SNP, or deionized water (control) for 24 h and then were exposed to 5°C for 48 h. Data are represented as mean ± SD (*n* = 3). Different letters indicate a significant difference between samples according to Duncan's new multiple range test (*p* < 0.05).

To explore whether MT alleviates oxidative damage from cold stress and whether NO is involved in the process of MT-induced antioxidation under cold stress, we examined ROS accumulation and antioxidant system activity in stressed seedlings pretreated with 100 μM MT, 75 μM SNP, 100 μM cPTIO + 100 μM MT, 50 μM p-CPA + 75 μM SNP, or deionized water. The results showed that MT- and SNP-treated seedlings exhibited far fewer damage symptoms caused by cold stress (Figure 8A). The mitigation of MT in chilling injury of cucumber seedlings was weakened by cPTIO, a specific scavenger of NO. However, the MT synthetic inhibitor p-CPA had little effect on NO-induced alleviation of chilling injury. Figures 8B–D show that cold stress significantly increased the generation of H_2_O_2_ and O_2_^−^, and as a result of excess ROS accumulation, the stressed seedlings revealed lipid peroxidation, as evidenced by increased EL (Figure 8E). Exogenous MT or SNP dramatically decreased the accumulation of H_2_O_2_ and O_2_^−^ and markedly reduced EL in cucumber seedlings under cold stress. MT-induced decreases in the accumulation of H_2_O_2_ and O_2_^−^ and EL were blocked by cPTIO, but the decrease caused by SNP in stressed seedlings was not affected by p-CPA. These data suggest that MT-induced cold tolerance is connected with increased ROS scavenging activity. To test the speculation that NO may be involved in MT-induced antioxidant activities, we determined the changes in the antioxidant system in cucumber seedlings after exposure to 5°C for 48 h. As shown in Figures 9A–D, cold significantly increased in the activities of SOD, POD, APX, and GR in cucumber seedlings. Compared with H_2_O-treated seedlings, the activities of SOD, POD, APX, and GR in MT-treated seedlings increased by 6.9, 29.1, 31.6, and 29.4%, respectively, and those in SNP-treated seedlings increased by 5.0, 26.9, 21.7, and 36.0%, respectively. cPTIO significantly repressed MT-induced activities of antioxidant enzymes (*p* < 0.05), but p-CPA showed little effect on NO-induced activities of antioxidant enzymes. Cold stress also increased the mRNA abundances of *SOD, POD, APX*, and *GR*, and this increase was markedly greater in MT and SNP treatments. Compared to MT treatment alone, cPTIO + MT treatment resulted in significant reduction of the mRNA abundances of *SOD, POD, APX*, and *GR*. However, p-CPA + SNP treatment caused no remarkable difference in the mRNA abundances of *SOD, POD, APX*, and *GR* relative to SNP treatment (Figures 9E–H). We also found that the contents of AsA and GSH were significantly decreased in H_2_O-treated seedlings after exposure to 5°C for 48 h. Seedlings pretreated with MT and SNP showed dramatically higher AsA and GSH contents than H_2_O-treated seedlings (*p* < 0.05) during cold stress. With the application of cPTIO, the positive effect of MT on the generation of GSH and AsA was obviously blocked; however, p-CPA did not inhibit the positive effect of SNP on GSH and AsA generation. Similarly, cold stress led to a significant decrease in GSH/GSSG and AsA/DHA. Both MT and SNP treatments showed higher GSH/GSSG and AsA/DHA than the H_2_O treatment under cold stress (*p* < 0.05). cPTIO markedly reduced the effect of MT on GSH/GSSG and AsA/DHA, while p-CPA showed little influence on the SNP-induced regulation of GSH/GSSG and AsA/DHA (Supplementary Figure 1). These results indicate that MT and SNP can scavenge ROS by increasing the activity of antioxidant enzymes the contents of AsA and GSH, as well as GSH/GSSG and AsA/DHA, alleviating oxidative damage caused by cold stress in cucumber seedlings. NO may function as a downstream signal of MT and play a role in ROS scavenging.

### The Role of NO in MT-Induced CO_2_ Assimilation

**Figure 10:**
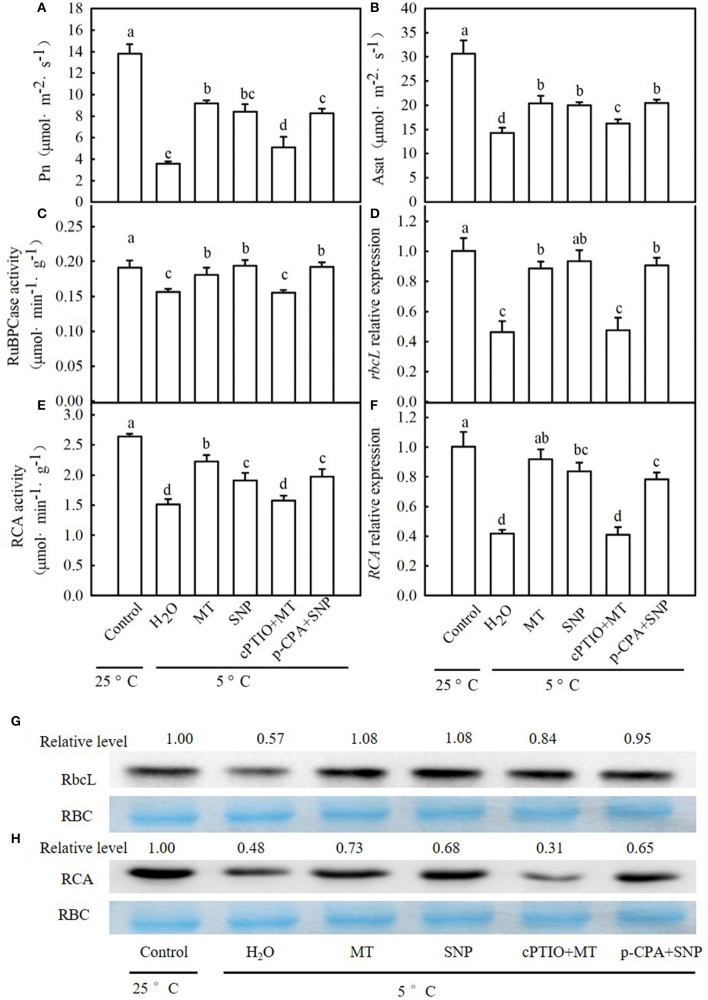
Interactive effects of MT and SNP on *P*_n_, A_sat_, activity of Ribulose-1,5-bisphosphate carboxylase (RuBPCase) and Rubisco activase (RCA), and relative expression of *rbcL* and *RCA* in mRNA and the protein level in cucumber seedlings under cold stress. **(A)** *P*_n_, **(B)** A_sat_; **(C,D)** activity of RuBPCase and RCA, respectively; **(E,F)** Relative mRNA expression of *rbcL* and *RCA*, respectively; and **(G,H)** Protein level of *rbcL* and *RCA*, respectively. Coomassie Brilliant Blue staining Rubisco (RBC) protein is shown for equal loading control. Western blotting was performed three times with three independent biological samples, and similar results were obtained. Three-leaf stage seedlings were foliar sprayed with 100 μM MT, 75 μM SNP, 100 μM cPTIO+100 μM MT, 50 μM p-CPA+75 μM SNP, or deionized water (control) for 24 h and then were exposed to 5°C for 24 h. Data are represented as mean ± SD (*n* = 3). Different letters indicate a significant difference between samples according to Duncan's new multiple range test (*p* < 0.05).

When seedlings were subjected to chilling for 24 h, *P*_n_ and A_sat_ in the H_2_O treatment were decreased by 74.2 and 53.0% (Figures 10A,B), respectively, in comparison to those of the control. MT- and SNP-treated seedlings exhibited a significant increase in *P*_n_ and A_sat_ relative to H_2_O-treated seedlings under cold stress. The increases in *P*_n_ and A_sat_ in MT treatment were weakened by cPTIO, whereas those in NO-treated seedlings were not distinctly affected by p-CPA. After exposure to 5°C for 24 h, the activities of RuBPCase and RCA also distinctly decreased in all treatments (Figures 10C,E). Compared to H_2_O treatment, MT and SNP increased the RuBPCase and RCA activity under cold stress (*p* < 0.05). The application of cPTIO significantly weakened MT-induced activities of RuBPCase and RCA, but p-CPA did not produce a noticeable effect on NO-induced RuBPCase or RCA activity. Similarly, relative expression at the mRNA and protein levels of *rbcL* in H_2_O-treated seedlings was decreased by 53.8 and 43.0%, respectively (Figures 10D,G), and that of *RCA* in H_2_O-treated seedlings was reduced by 58.5 and 52.0%, respectively (Figures 10F,H), relative to those of the control. MT and SNP treatments showed significantly higher relative expression of *rbcL* and *RCA* at both the mRNA and protein levels than H_2_O treatment (*p* < 0.05). The upregulation in relative expression of *rbcL* and *RCA* in MT treatment was also severely curtailed by cPTIO while that in SNP-treated seedlings was not affected by p-CPA.

### The Role of NO in MT-Induced Photoprotection

To explore whether exogenous MT is relevant to photoprotection, we compared the degree of photoinhibition at photosystem I (PSI) and PSII in different treatment seedlings under cold stress. Both MT and SNP significantly increased *F*_v_/*F*_m_ and *Φ*_PSII_ relative to H_2_O treatment (*p* < 0.05) during cold stress. The increases in *F*_v_/*F*_m_ and *Φ*_PSII_ in MT treatment were markedly weakened by cPTIO, but those in SNP treatment were not remarkably influenced by p-CPA (Supplementary Figures 2A,B). In addition, histochemical observations with an inverted fluorescence microscope for *F*_v_/*F*_m_ and *Φ*_PSII_ agreed with the detected results. From the chlorophyll fluorescence transients of dark-adapted leaves, we found that the chlorophyll transients fluorescence showed significant modifications when seedlings were subjected to 5°C for 24 h (Supplementary Figure 2C). Cold-stressed leaves revealed an increase in *F*_0_ **(O)** while revealing a decrease in *F*_m_ (IP) relative to the control leaves. The values of *F*_0_ were significantly lower, but those of *F*_m_ were dramatically higher in MT-, SNP-, and p-CPA + SNP-treated seedlings than in H_2_O-treated seedlings (*p* < 0.05); no differences were observed in *F*_0_ and *F*_m_ between cPTIO + MT- or H_2_O-treated seedlings. Cold stress led to an increase in variable fluorescence (*F*_v_) of the O-J part but resulted in a decrease in the J-I and I-P phases. To avoid the production of any interference and heterogeneity, owing to different *F*_0_ and *F*_m_, we performed normalization at *F*_m_. As shown in Supplementary Figure 2D, the increase in the O-J phase decreased in MT-, SNP-, and p-CPA + SNP-treated seedlings than in H_2_O-treated seedlings, whereas those in cPTIO + MT-treated seedlings showed no significant difference relative to H_2_O-treated seedlings. Step J (2 ms) is a specific sign of limited electron transport for Q_A_ to Q_B_ and D1 protein damage (Liu et al., 2020). The Δ*V*_t_ curve showed that MT and SNP significantly decreased the amplitude of step J relative to H_2_O treatment. The decrease in step J in MT treatment was blocked by cPTIO, but p-CPA showed little effect on the SNP-induced reduction in the amplitude of step J (Supplementary Figure 2E). Supplementary Figure 2F reveals that cold stress reduced PI_ABS_ in cucumber seedlings (*p* < 0.05), and the decrease in PI_ABS_ of seedlings following 24 h stress was 98.5, 71.7, 61.0, 94.7, and 61.8% in the H_2_O, MT, SNP, cPTIO + MT, and p-CPA + SNP treatment, respectively, compared with the control. Cold stress resulted in a significant decrease in Ψ_0_ but an obvious increase in *Φ*_D0_ (Supplementary Figures 2G,H). Compared with H_2_O treatment, a decrease in Ψ_0_ and an increase in *Φ*_D0_ were significantly attenuated in MT and SNP treatments (*p* < 0.05). MT-induced variations in Ψ_0_ and *Φ*_D0_ were repressed by cPTIO, whereas SNP-induced variations in Ψ_0_ and *Φ*_D0_ were only marginally affected by p-CPA.

To validate the above results and to further explore the effect of exogenous MT on photoprotection in response to chilling and the role of NO in MT-induced photoprotection, we analyzed the protein levels of D1 and PsaD in stressed seedlings. Cold stress largely downregulated the protein levels of D1 and PsaD in all treatment seedlings (Supplementary Figure 2I). In comparison to H_2_O-treated seedlings, MT- and SNP-treated seedlings displayed a significantly higher accumulation of D1 and PsaD proteins after exposure to cold stress for 24 h. On the application of cPTIO, the positive effect of MT on the accumulation of D1 and PsaD proteins was blocked, while p-CPA did not inhibit the effect of SNP on D1 or PsaD protein levels. As shown in Supplementary Figure 2J, we found that Δ*I*/*I*_0_ was decreased by 67.2% in H_2_O-treated seedlings when exposed to 5°C for 24 h. The decrease in Δ*I*/*I*_0_ in MT and SNP treatments was significantly less than that in H_2_O treatment, indicating that MT and SNP play a positive role in protecting the PSI reaction center against photodamage caused by cold stress. cPTIO + MT-treated seedlings displayed an evidently lower Δ*I*/*I*_0_ than MT-treated seedlings (*p* < 0.05), but p-CPA + SNP-treated seedlings showed little difference in Δ*I*/*I*_0_ compared with SNP-treated seedlings under cold stress. These data suggest that MT and NO alleviated the inhibition of photosynthetic electron transport and the damage to D1 protein and the PSI reaction center caused by cold stress. NO acts as a downstream signal of MT and plays an important role in the process.

### MT and SNP Upregulate the Expression of Cold-Responsive Genes Under Cold Stress

**Figure 11:**
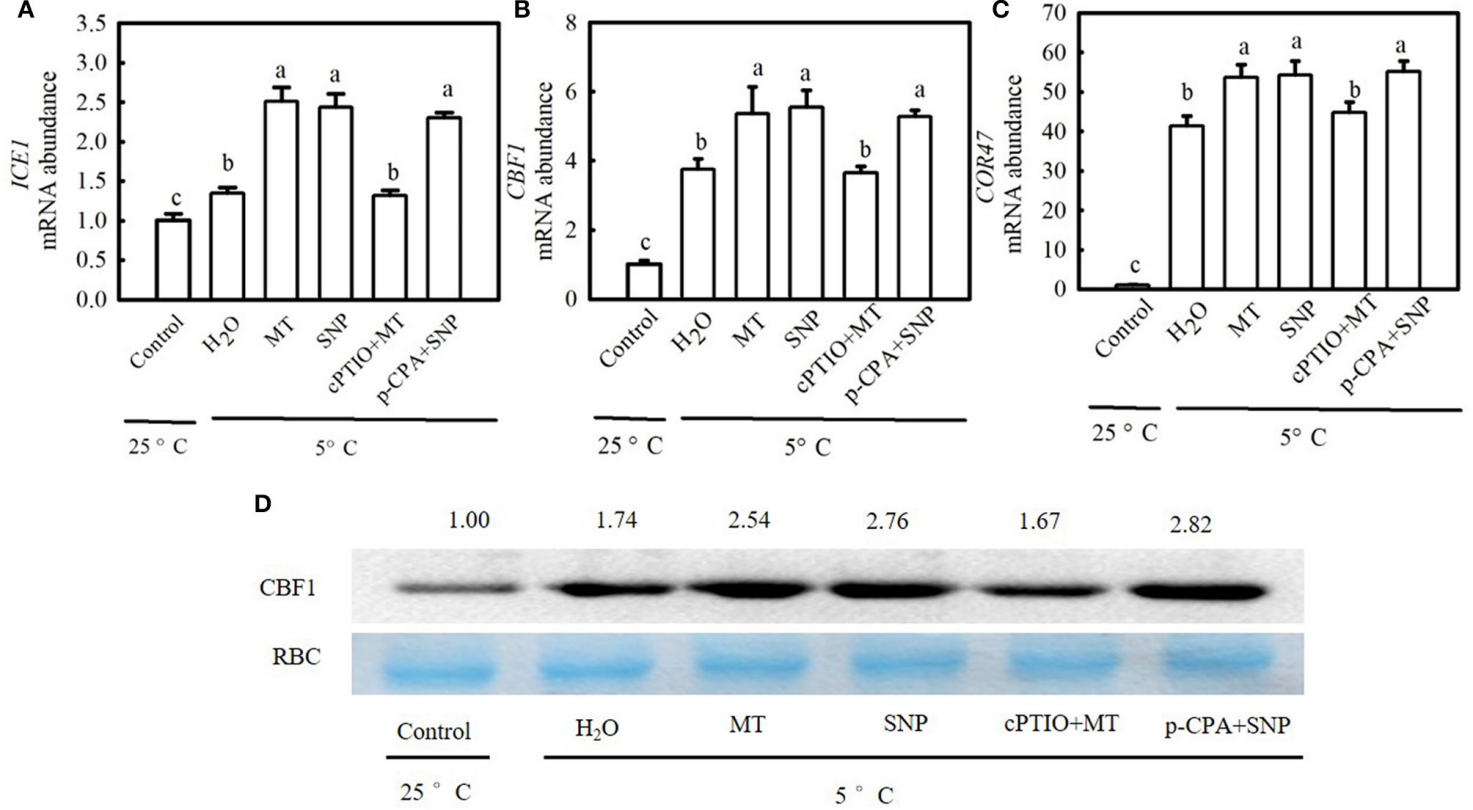
Interactive effects of MT and SNP on the relative mRNA expression of cold-responsive (*COR47*) genes and the protein level of C-repeat-binding factor (*CBF1*) in cucumber seedlings under cold stress. **(A–C)** Relative mRNA expression of inducer of *CBF* expression (*ICE1*), *CBF1*, and *COR47*, respectively. **(D)** *CBF1* protein level. Three-leaf stage seedlings were foliar sprayed with 100 μM MT, 75 μM SNP, 100 μM cPTIO + 100 μM MT, 50 μM p-CPA + 75 μM SNP, or deionized water (control) for 24 h and then exposed to 5°C for 24 h. Data are represented as mean ± SD (*n* = 3). Different letters indicate a significant difference between samples according to Duncan's new multiple range test (*p* < 0.05).

To examine whether the ICE1–CBF–COR transcriptional cascade contributed to MT- and SNP-induced cold tolerance in cucumber seedlings, we compared the responses of the *CBF1*, an inducer of *CBF* expression (*ICE1*), and cold-responsive (*COR47*) genes to cold stress among different treatments. Figure 11 shows that cold stress largely upregulated relative mRNA expression of *ICE1, CBF1*, and *COR47* as well as CBF1 protein accumulation in stressed seedlings, which was more obvious in MT and SNP treatments than in H_2_O treatment. For example, relative mRNA expression of *CBF1* in MT- and SNP-treated seedlings increased by 4.3- and 4.5-fold, respectively, but that in H_2_O-treated seedlings only increased by 2.8-fold after exposure to 5°C for 48 h. The treatment with cPTIO significantly weakened the positive effect of MT on the expression of *COR47* genes, whereas the application of p-CPA showed little effect on NO-induced regulation of the expression of the above genes.

## Discussion

In the present study, we found that the endogenous MT emission system was activated by cold stress in the “JY35” and “ZN6” cucumber seedlings. The activities of TDC and ASMT, relative mRNA expression of *TDC* and *ASMT*, and MT accumulation in the “JY35” seedlings were significantly higher than those in the “ZN6” seedlings whereas 2-hydroxymelatonin accumulation in the “JY35” seedlings was markedly lower than that in the “ZN6” seedlings (*p* < 0.05) during cold stress (Figure 1). The “JY35” seedlings also exhibited higher *F*_v_/*F*_m_ and *Φ*_PSII_, lower MDA, EL, and CI, and minor damage than the “ZN6” seedlings following cold stress (Figure 2). These data suggest that endogenous MT accumulation under cold stress is positively correlated with cold tolerance in different varieties of cucumber seedlings.

Lei et al. (2004) first reported that exogenous MT attenuated cold-induced apoptosis in carrot suspension cells probably through upregulating polyamine levels. Afterward, Posmyk et al. (2009) revealed that MT alleviated lipid peroxidation in plant cell membranes caused by cold stress, but excessive MT concentration in cucumber seeds provoked oxidative changes in proteins. Heterologous human *SNAcT* expression in rice promoted the accumulation of endogenous MT, and the transgenic rice seedlings showed elevated chlorophyll synthesis and greater cold tolerance (Kang et al., 2010). Exogenous MT also induces the expression of *COR47* factors, such as *CBFs, DREBs*, and *COR15a* (Bajwa et al., 2014), indicating that MT has the physiological function of responding to cold stress and transcriptional activation of related metabolic processes. Seed pretreated with MT seemed to limit H_2_O_2_ accumulation during germination under cold stress and recovery periods (Bałabusta et al., 2016). The present study revealed that exogenous MT is involved in the regulation of cold tolerance in a concentration-dependent manner, as 100 μM MT increased *F*_v_/*F*_m_, *Φ*_PSII_, and *P*_n_, decreased MDA accumulation and EL, and alleviated foliar damage in cucumber seedlings caused by cold stress; however, a higher concentration inhibited these positive effects (Figure 3).

Zhang et al. (2019) found that NR-derived NO synthesis was also involved in chilling acclimation and freezing tolerance in forage legumes. Liu et al. (2015) illustrated that exogenous MT treatment increased NO accumulation in tomato roots under alkaline stress, and MT-induced NO production was accompanied by improvement of alkaline tolerance. Kaya et al. (2019) also observed that MT increased the tolerance of wheat seedlings to Cd toxicity by promoting endogenous NO. Tolerance to iron deficiency and salt stress induced by MT in pepper may be involved in downstream signal crosstalk between NO and H_2_S (Kaya et al., 2020a). Our present data indicated that the endogenous NO emission system was also induced by cold stress (Figure 5), and 100 μM MT significantly increased NR activity and relative mRNA expression, as well as NO accumulation in cucumber seedlings (Figure 4). However, no significant differences were observed among SNP, p-CPA, and control treatments in MT content and relative mRNA expression of the key genes of MT biosynthesis (Figure 7). Both MT and SNP markedly enhanced cold tolerance, and the *F*_v_/*F*_m_ and *Φ*_PSII_ reached maximum, but EL reduced to the minimum value under 100 μM MT or 75 μM SNP treatments relative to the control and with other concentrations of MT or SNP (Figures 4, 6). The addition of cPTIO reduced MT-induced cold tolerance, whereas NO-induced cold tolerance was not affected by p-CPA (Figure 8). By detecting the antioxidant system, photosynthetic carbon assimilation, photoprotection, and the ICE–CBF–COR pathway, we speculated that NO, as a downstream signal, plays a crucial role in MT-induced cold tolerance in cucumber seedlings.

Generally, cell membrane dysfunction and excessive ROS production are the two main events of chilling injury (Chongchatuporn et al., 2013). When ROS accumulation is higher than the level that the defense mechanism can handle, cells experience oxidative stress (Schippers et al., 2016). Previous studies have demonstrated that MT and NO act as antioxidative signaling molecules to defend against abiotic stress by declining ROS production and enhancing the activity of antioxidative enzymes (Lei et al., 2004; Siddiqui et al., 2011; Bałabusta et al., 2016; Nawaz et al., 2018; Yan et al., 2019). Under arsenic (As) toxicity conditions, the application of Mel and Ca^2+^ synergistically suppressed the programmed cell death features of plants (nuclear condensation and nuclear fragmentation) in guard cells of stomata, DNA damage, and the formation of ROS in guard cells, leaves, and roots (Siddiqui et al., 2020). Here, we confirmed that cold stress triggered a burst of H_2_O_2_ and O_2_^−^, enhanced El and MDA accumulation, and subsequently caused ROS-associated injury in cucumber seedlings. Exogenous MT and SNP distinctly decreased ROS accumulation and ROS-induced damage in cucumber seedlings during cold stress (Figure 8). Furthermore, MT and SNP also increased the activity of antioxidant enzymes and their relative mRNA expression (Figure 9), suggesting that they can protect the cellular membrane and subcellular structures of cucumber under cold.

Ascorbic acid and GSH, the key cellular redox signal elements, are usually used for both ROS detoxification and redox signal transmission. In the AsA–GSH cycle, reduced ascorbate (AsA) is oxidized to unstable radical monodehydroascorbate (MDHA), and MDHA rapidly generates dehydroascorbate (DHA) (Smirnoff, 2000). Subsequently, DHA is converted back to AsA with the usage of reduced GSH as the electron donor (Fotopoulos et al., 2010). It is believed that maintaining higher proportions of AsA/DHA and GSH/GSSG is very important to ensure the normal participation of AsA and GSH in the AsA–GSH cycle and other physiological processes under abiotic stress. Therefore, higher proportions of AsA/DHA and/or GSH/GSSG might be the key factors for effective protection against abiotic stress-induced ROS accumulation (Fotopoulos et al., 2010; Bałabusta et al., 2016). Kaya et al. (2020b,c) found that NO upregulated the activities of the AsA–GSH cycle and antioxidant enzymes, so it may play a central role as a signaling molecule in salt and Cd tolerance in pepper plants. Here, we found that cold-stressed cucumber seedlings exhibited a significant decrease in AsA, GSH, AsA/DHA, and GSH/GSSG. The application of MT and SNP notably enhanced AsA, GSH, AsA/DHA, and GSH/GSSG under cold stress, compared with H_2_O-pretreated seedlings (Supplementary Figure 1). These results indicate that cold stress seriously influences the redox balance in cucumber seedlings. Exogenous MT and SNP maintain redox states by upregulating the proportion of AsA/DHA and GSH/GSSG and consequently balance ROS generation and scavenging. To further elucidate the interactive role of MT and NO in exerting effective protection against chilling-induced ROS accumulation, we also applied p-CPA and cPTIO. The positive effect of MT on the antioxidant ability during cold stress was distinctly suppressed by cPTIO, but that of SNP on NO-induced ROS scavenging capacity was not affected by p-CPA. These data indicate that NO is involved in cell membrane protection induced by MT, especially in defense against oxidative stress during chilling.

Recently, some findings on the function of MT in plant photosynthesis have been published. Liang et al. (2019) reported that MT upregulates the genes involved in CO_2_ fixation, such as *PGK, FBA*, and *FBP*, in kiwifruit seedlings. MT also improves photosynthetic capacity by inhibiting stomatal closure, enhancing light energy absorption, promoting electron transport in PSII, and increasing the adaptability of kiwifruit seedlings to drought stress. A similar result illustrated that the application of MT notably increased photosynthesis efficiency, upregulated chlorophyll synthesis genes, and PSII maximum efficiency (*F*_v_/*F*_m_) in nickel-stressed tomato plants (Jahan et al., 2020). Siddiqui et al. (2020) confirmed that MT enhanced gas exchange parameters and the activity of enzymes involved in the photosynthesis process (carbonic anhydrase and RuBisco) and Chl biosynthesis (δ-aminole vulinic acid dehydratase) and decreased the activity of Chl degrading enzyme (chlorophyllase) under As toxicity conditions. Similarly, Tan et al. (2008) revealed that NO also increased the photosynthesis of wheat seedlings subjected to osmotic stress. In cucumber seedlings, however, a cross-talk between MT and NO in the regulation of photosynthesis under cold stress is unclear. In the present study, we observed that both MT and SNP markedly increased *P*_n_, A_sat_, activities of RuBPCase and RCA, relative mRNA expression of *rbcL* and *RCA*, and the protein accumulation of rbcL and RCA, compared with H_2_O treatment under cold stress. cPTIO substantially abolished MT promotion in CO_2_ assimilation during chilling, whereas p-CPA did not inhibit the function of SNP under stress conditions (Figure 10). Meanwhile, MT- and SNP-treated seedlings showed a significantly increased *F*_v_/*F*_m_, *Φ*_PSII_, PI_ABS_, ψ_0_, and D1 protein, as well as ΔI/I_0_ and PsaD accumulation, relative to H_2_O-treated seedlings under cold stress (Supplementary Figure 2). These data support the view that MT and NO play a positive role in the photoprotection for both PSII and PSI in cucumber seedlings. The D1 protein repair pathway is considered to be conducive to cold tolerance in plants (Fang et al., 2019; Liu et al., 2020). In addition, PsaD plays distinct roles in facilitating ferredoxin-mediated NADP^+^ photoreduction on the reducing side of PSI (Chitnis et al., 1996). From this, it seems to be clear that MT- and NO-induced photoprotection may be involved in the activation of the D1 repair pathway in PSII and improvement of ferredoxin-mediated NADP^+^ photoreduction in PSI during cold stress. Considering our investigation after the application of cPTIO and p-CPA, the results suggest that NO is required as a downstream signal for MT-induced CO_2_ assimilation and photoprotection in cucumber seedlings.

In higher plants, the ICE1–CBF–COR transcriptional cascade is considered the most well-understood cold acclimation signaling pathway. In this pathway, CBFs are crucial transcription factors (Zhou et al., 2018) and are essential for the induction of cold tolerance (Kang et al., 2013). Thomashow (2001) reported that the overexpression of *CBF* genes increased freezing tolerance in Arabidopsis whereas the knockdown of *CBF1* and/or *CBF3* decreased plant tolerance to freezing stress after cold acclimation (Novillo et al., 2007). Previous studies have demonstrated that hormones play a crucial role in the response of plants to cold stress. For instance, a decrease in endogenous ethylene weakens the transcriptional inhibition of *CBFs* by ethylene signaling and triggers *CBF*-dependent cold acclimation during the early stages of cold stress. Subsequently, the cold stress response promotes EIN3 accumulation to prevent overaccumulation of *CBFs* through feedback adjustment (Shi Y. T. et al., 2015). The present results revealed that cold stress increased the relative mRNA levels of *ICE1, CBF1*, and *COR47*, and CBF1 protein accumulation in cucumber seedlings (Figure 11). MT and SNP significantly upregulated the expression of *ICE1, CBF1*, and *COR47*, indicating that MT and NO are involved in the ICE1–CBF–COR transcriptional cascade. The application of cPTIO repressed MT-induced positive effect on the transcriptional and protein levels of *ICE1, CBF1*, and *COR47*, whereas p-CPA showed little influence on SNP-induced regulation of the expression of *COR47* genes. These results further illustrate that NO functions as a downstream signal to participate in MT-induced cold tolerance in cucumber seedlings.

## Conclusions

**Figure 12:**
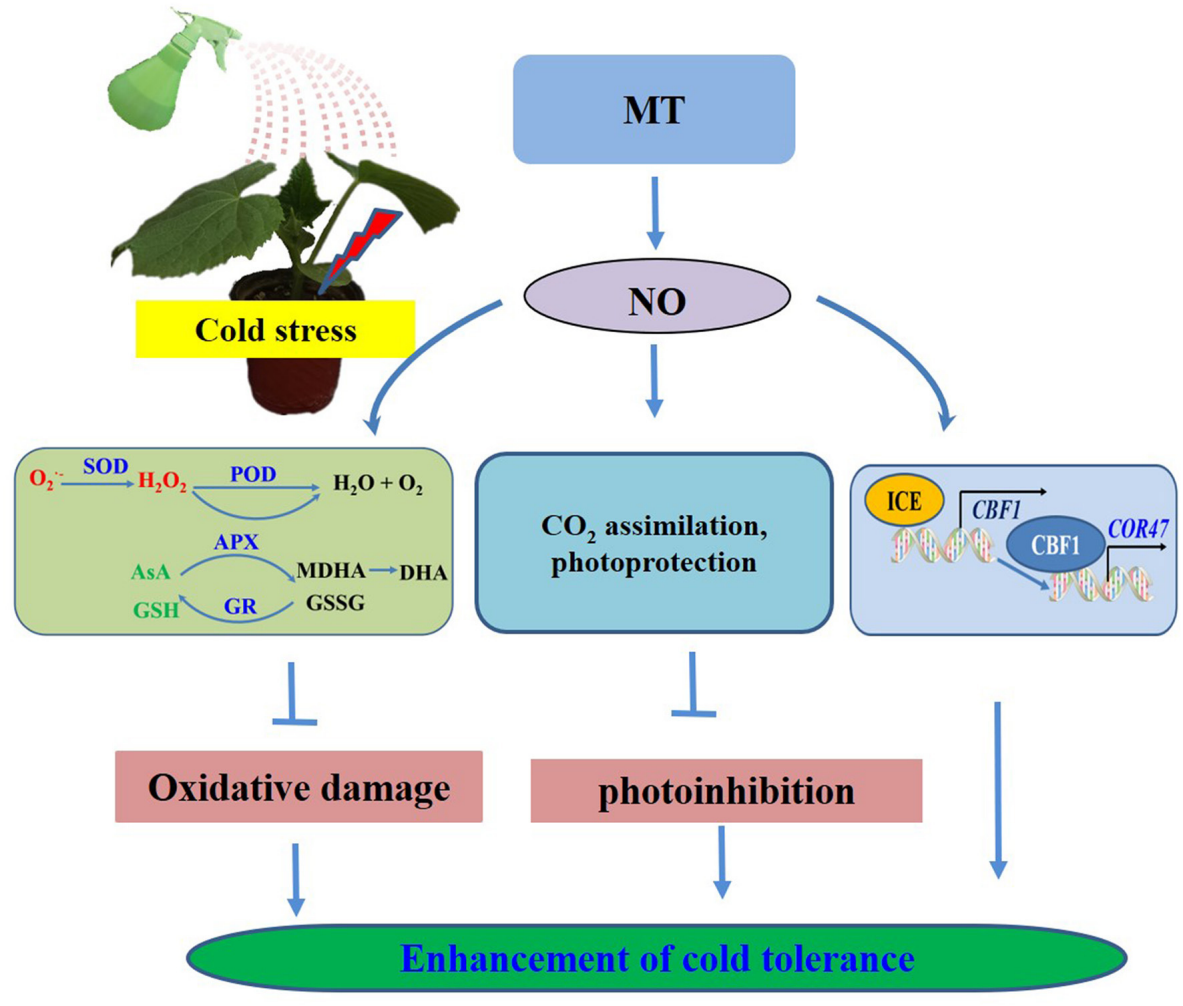
Simplified schematic model for MT-induced cold tolerance in cucumber.

Based on the present results, we proposed a model for MT-induced cold tolerance in cucumber seedlings. As shown in Figure 12, endogenous MT induced by cold or the application of MT and SNP alleviated the negative effects of growth and photosynthesis by scavenging excessive ROS, by protecting the photosynthetic apparatus and activating the ICE–CBF1–COR signaling pathway. All the investigated results indicate that NO, acting as a downstream signal, plays a critical role in MT-induced cold tolerance in cucumber seedlings. A number of evidence for the importance of exogenous MT and SNP provide potential new approaches for relieving chilling injury in cold-sensitive plants. To better reveal the detailed mechanisms and interaction of MT- and NO-induced cold tolerance in plants, further studies using advanced molecular techniques and mutant analyses are required.

## Data Availability Statement

The original contributions presented in the study are included in the article/Supplementary Material, further inquiries can be directed to the corresponding author/s.

## Author Contributions

YF performed most part of the experiment, analyzed the data, and completed the first draft. XA and HB designed the research and edited the manuscript. XF, LH, CX, and CL worked together with YF to accomplish the experiment. All authors contributed to the article and approved the submitted version.

## Conflict of Interest

The authors declare that the research was conducted in the absence of any commercial or financial relationships that could be construed as a potential conflict of interest. The reviewer SZ declared a shared affiliation, with no collaboration, with the authors to the handling editor at the time of the review.

## Publisher's Note

All claims expressed in this article are solely those of the authors and do not necessarily represent those of their affiliated organizations, or those of the publisher, the editors and the reviewers. Any product that may be evaluated in this article, or claim that may be made by its manufacturer, is not guaranteed or endorsed by the publisher.
