# Supplementary material for: Nitric Oxide Functions as a Downstream Signal for Melatonin-Induced Cold Tolerance in Cucumber Seedlings
Source: Front Plant Sci. 2021 Jul 23;12:686545. doi: 10.3389/fpls.2021.686545 (PMC8343141; doi:10.3389/fpls.2021.686545)
Supplement: Supplementary file 1 [file Data_Sheet_1.PDF]

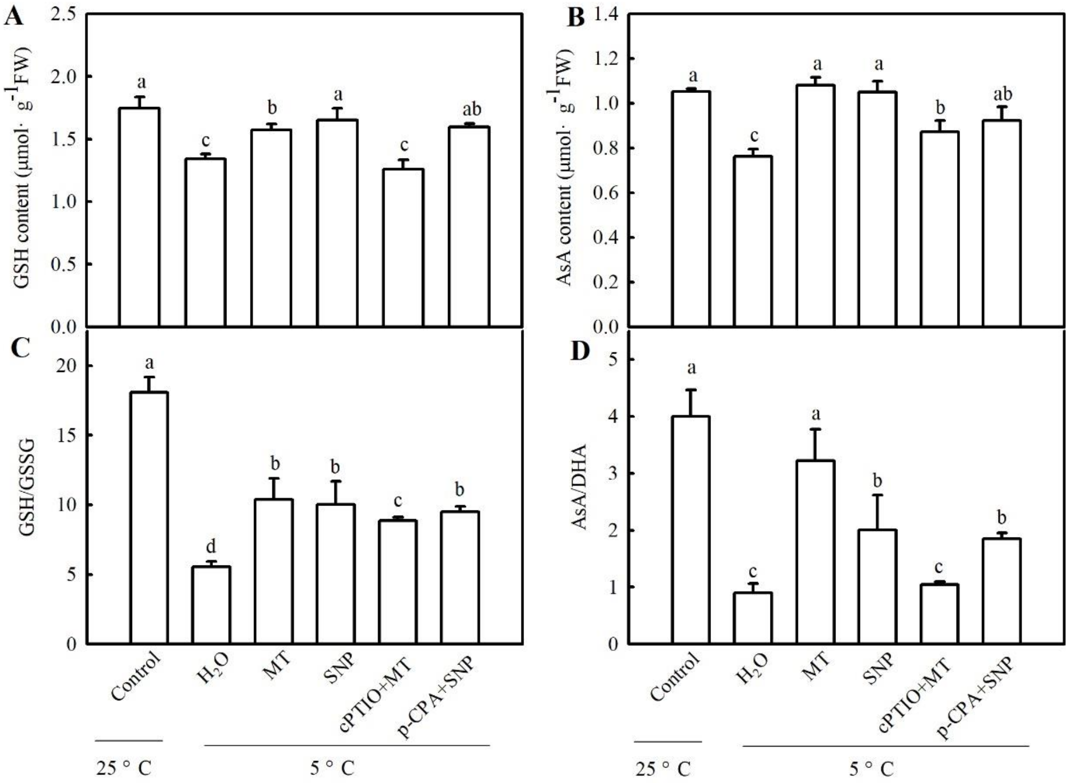

Fig. S. 1 Interactive effects of MT and SNP on the contents GSH, AsA, GSH/GSSG, and AsA/DHA in cucumber seedlings under cold stress. **(A)** GSH content, **(B)** AsA content, **(C)** GSH/GSSG, **(D)** AsA/DHA. Three-leaf stage seedling were foliar sprayed with 100 μM MT, 75 μM SNP, 100 μM cPTIO+100 μM MT, 50 μM p-CPA+75 μM SNP, or deionized water (control), respectively for 24 h, and then were exposure to 5 °C for 48 h. Data are the mean ± SD (n = 3). Different letters indicate differ significantly between samples according to Duncan's new multiple range test ($P < 0.05$).

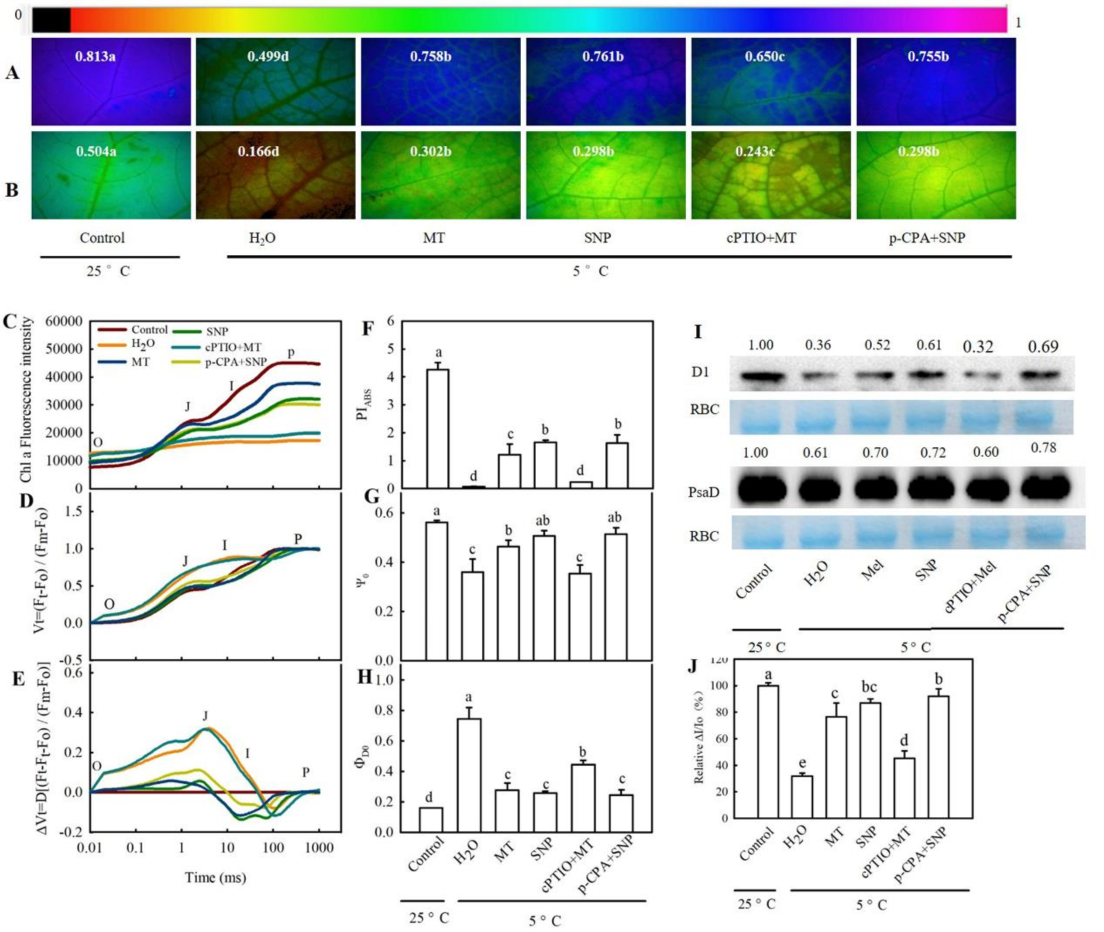

Fig. S. 2 Interactive effects of MT and SNP on photoprotection in cucumber seedlings under cold stress. Three-leaf stage seedling were foliar sprayed with 100 μM MT, 75 μM SNP, 100 μM cPTIO+100 μM MT, 50 μM p-CPA+75 μM SNP, or deionized water (control), respectively for 24 h, and then were exposure to 5 °C for 24 h. **(A)** F_v/F_m; **(B)** Φ_{PSII}. The false color code depicted at the top of the image ranging from 0 (black) to 1.0 (purple) represents the degree of photoinhibition at PSII. **(C)** OJIP curve; **(D)** V_t; **(E)** ΔV_t; **(F)** PI_{ABS}; **(G)** ψ_0; **(H)** φ_{D0}; **(I)** D1 and PsaD proteins. Value above each line are the relative accumulation of the protein; **(J)** $\triangle I/I_0$. Data are the mean ± SD (n = 3). Different letters indicate differ significantly between samples according to Duncan's new multiple range test ($P < 0.05$).
